# INB^3^P: A Multi‐Modal and Interpretable Co‐Attention Framework Integrating Property‐Aware Explanations and Memory‐Bank Contrastive Fusion for Blood–Brain Barrier Penetrating Peptide Discovery

**DOI:** 10.1002/advs.202523984

**Published:** 2026-04-03

**Authors:** Jingwei Lv, Qianyang Wu, Jian Liu, Binlu Yang, Yuanhao Li, Junlin Xu, Yajie Meng, Leyi Wei, Zilong Zhang, Quan Zou, Xiulai Li, Feifei Cui

**Affiliations:** ^1^ School of Computer Science and Technology Hainan University Haikou China; ^2^ School of Computer Science and Technology Wuhan University of Science and Technology Wuhan Hubei China; ^3^ School of Computer Science and Artificial Intelligence Wuhan Textile University Wuhan Hubei China; ^4^ Centre For Artificial Intelligence‐Driven Drug Discovery Faculty of Applied Science Macao Polytechnic University Macao SAR China; ^5^ Institute of Fundamental and Frontier Sciences University of Electronic Science and Technology of China Chengdu China

**Keywords:** contrastive learning, cns drug delivery, interpretability, multi‐modal deep learning, physicochemical‐guided augmentation

## Abstract

Functional peptide discovery, particularly for blood–brain barrier‐penetrating peptides (BBBPPs), is strictly limited by extreme data scarcity and the “black‐box” nature of deep learning. Here, INB^3^P is presented as a physics‐informed, multi‐modal framework designed to address these challenges. Physicochemical‐guided mutagenesis (PCGM), a novel augmentation strategy that enforces biochemical constraints to expand training diversity without violating the biological manifold. INB^3^P integrates PCGM with a bi‐directional co‐attention mechanism fusing sequence and structure, optimized via contrastive learning and a Stable‐MCC loss. INB^3^P significantly outperforms state‐of‐the‐art baselines on the same independent test set used in a prior study. Crucially, the model autonomously rediscovers known biophysical mechanisms—including amphipathic motifs and long‐range contact stabilization—providing strong in silico validation of its learned representations. This work establishes a generalizable paradigm for learning from small, imbalanced biological datasets. To facilitate community adoption, a web server is provided at http://www.bioai‐lab.com/INBP, featuring a standalone PCGM module, empowering researchers to apply physics‐guided augmentation strategy to their own sparse datasets.

## Graphical abstract

INB^3^P is a multimodal framework for blood–brain barrier‐penetrating peptide prediction under extreme data scarcity and class imbalance. By combining physicochemical‐guided augmentation, sequence–structure co‐attention, and imbalance‐aware optimization, it improves predictive performance and interpretability. Perturbation‐based faithfulness analysis further shows that highlighted residues meaningfully influence model outputs, supporting biologically informed peptide prioritization.

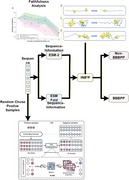

## 1 Introduction

Deep learning has revolutionized therapeutic peptide discovery [1], yet its application to specialized functional classes is severely hindered by two pervasive challenges: extreme data scarcity [2] and the “black‐box” interpretability gap [3]. Unlike generic protein language modeling, functional peptide discovery—such as identifying agents that traverse physiological barriers—often relies on small, heavily imbalanced datasets where verified positives are rare. In this regime, standard data augmentation strategies, such as random masking or sequence reversal, often risk generating biologically implausible sequences that dilute the mechanistic signal [4, 5]. Furthermore, end‐to‐end “black‐box” models frequently fail to elucidate the biophysical rules governing their predictions, leaving a critical gap between high predictive accuracy and actionable molecular design [6].

BBBPPs represent the archetype of this learning challenge. While they serve as essential modular shuttles for delivering therapeutics to the CNS [7], their discovery is impeded by a lack of structural conservation and a paucity of consistently annotated positives [8, 9]. BBBPPs do not form a single structural class; rather, they engage complex, multifaceted transcytosis pathways—ranging from receptor‐mediated (RMT) [10] to adsorptive‐mediated (AMT) [11] mechanisms—spanning wide and sometimes contradictory regimes of length, net charge, and hydrophobicity. Consequently, traditional predictors trained on handcrafted features often suffer from feature noise and fragile generalization [7], while recent deep‐learning approaches relying on purely generative augmentation (e.g., generative adversarial network (GANs)) struggle to maintain fidelity when the ground‐truth manifold is sparsely sampled [2].

**FIGURE 1:**
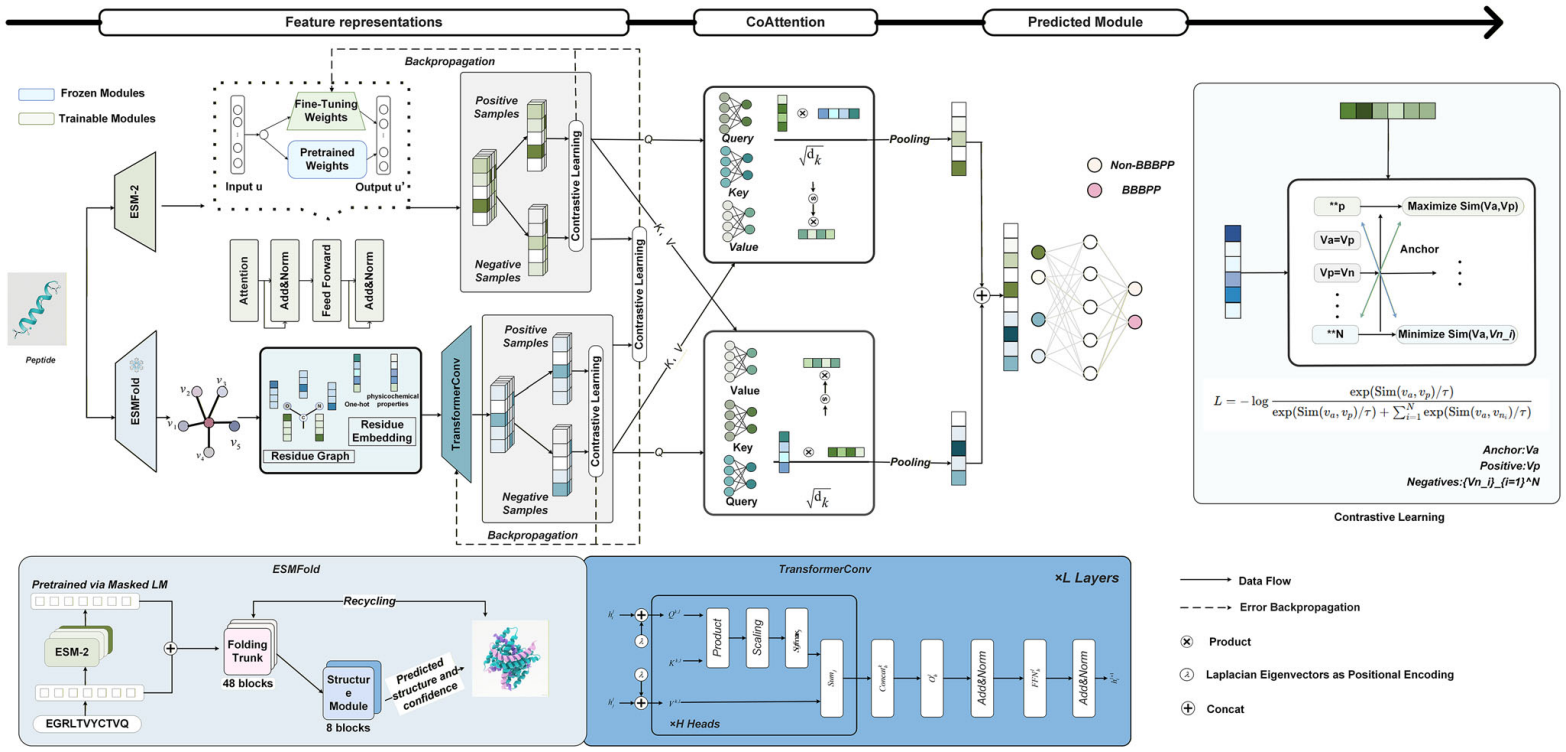
The architectural landscape of the INB^3^P framework. (Top) The end‐to‐end workflow integrates multi‐modal representations for robust BBBPP discovery. Peptide sequences are processed in parallel branches: the sequence modality is encoded by Evolutionary Scale Modeling 2 (ESM‐2) language model, while the structure modality is derived from Evolutionary Scale Modeling Fold (ESMFold) predictions and converted into residue‐level graphs enriched with physicochemical node features. These modalities are fused via a bi‐directional cross‐modal co‐attention mechanism, which aligns semantic tokens with structural neighborhoods. The network is trained using a two‐stage curriculum: Stage 1 utilizes contrastive learning (information noise‐contrastive estimation (InfoNCE) and supervised contrastive loss) to shape the latent space, while Stage 2 fine‐tunes the classifier using a composite imbalance‐robust objective (Focal Loss + Stable‐MCC). Blue and green blocks indicate frozen and trainable modules, respectively, highlighting the parameter‐efficient transfer learning strategy. (Bottom Left) Details of the ESMFold structure extraction module, which generates the spatial adjacency priors. (Bottom Right) The TransformerConv layer architecture used for graph reasoning, incorporating multi‐head attention and Laplacian positional encodings to capture long‐range structural dependencies.

To address these fundamental barriers, we introduce the integrative network for blood–brain–barrier‐penetrating peptides (INB^3^P, Figure 1), a physics‐informed, multi‐modal framework designed for robust learning under extreme scarcity. Distinct from prior task‐specific predictors, INB^3^P establishes a generalizable methodology for small‐sample biological sequence learning by integrating three core innovations:

1. PCGM: We propose a novel augmentation strategy that transcends simple sequence manipulation. By enforcing biochemical plausibility through property‐aware constraints (e.g., preserving charge–hydropathy balances), PCGM expands the training diversity without eroding the biological validity of the samples, offering a blueprint for augmenting other rare functional peptide classes.
2. Interpretable Cross‐Modal Fusion: We implement a bi‐directional co‐attention mechanism that unifies sequence semantics with predicted structural contexts. This architecture improves the interpretability of the learned representations by relating model decisions to residue‐level biophysical properties and putative design motifs.
3. Imbalance‐Robust Optimization: By coupling contrastive alignment with a differentiable Stable‐MCC objective, our framework directly optimizes decision boundaries for highly skewed distributions, ensuring sensitivity where conventional losses fail.

In this work, we demonstrate that INB^3^P not only outperforms state‐of‐the‐art baselines on the current BBBPP benchmark but also yields interpretability patterns that are broadly consistent with known biophysical characteristics of BBB‐penetrating peptides, despite the absence of explicit mechanistic supervision. The model's learned attention patterns align with established principles related to amphipathicity and electrostatic interaction, providing supportive in silico evidence that the framework captures biologically meaningful signals beyond purely superficial correlations. However, these findings should be interpreted within the scope of the current benchmark setting. INB^3^P thus offers a robust, interpretable route to peptide discovery that helps connect data‐driven prediction with mechanistic analysis.

Finally, to bridge the gap between computational modeling and experimental practice, we deploy INB^3^P as a publicly accessible web server. Unlike prior tools, our platform not only provides prediction scores but also visualizes residue‐level attention maps and structural contacts, thereby offering interpretable design cues. Furthermore, we democratize our data augmentation strategy by providing PCGM as a standalone tool on the server, enabling researchers in other domains to address data scarcity in their specific peptide discovery campaigns.

## 2 Results and Discussion

### 2.1 Statistical Characterization of the Dataset and Data‐Augmentation Framework

**FIGURE 2:**
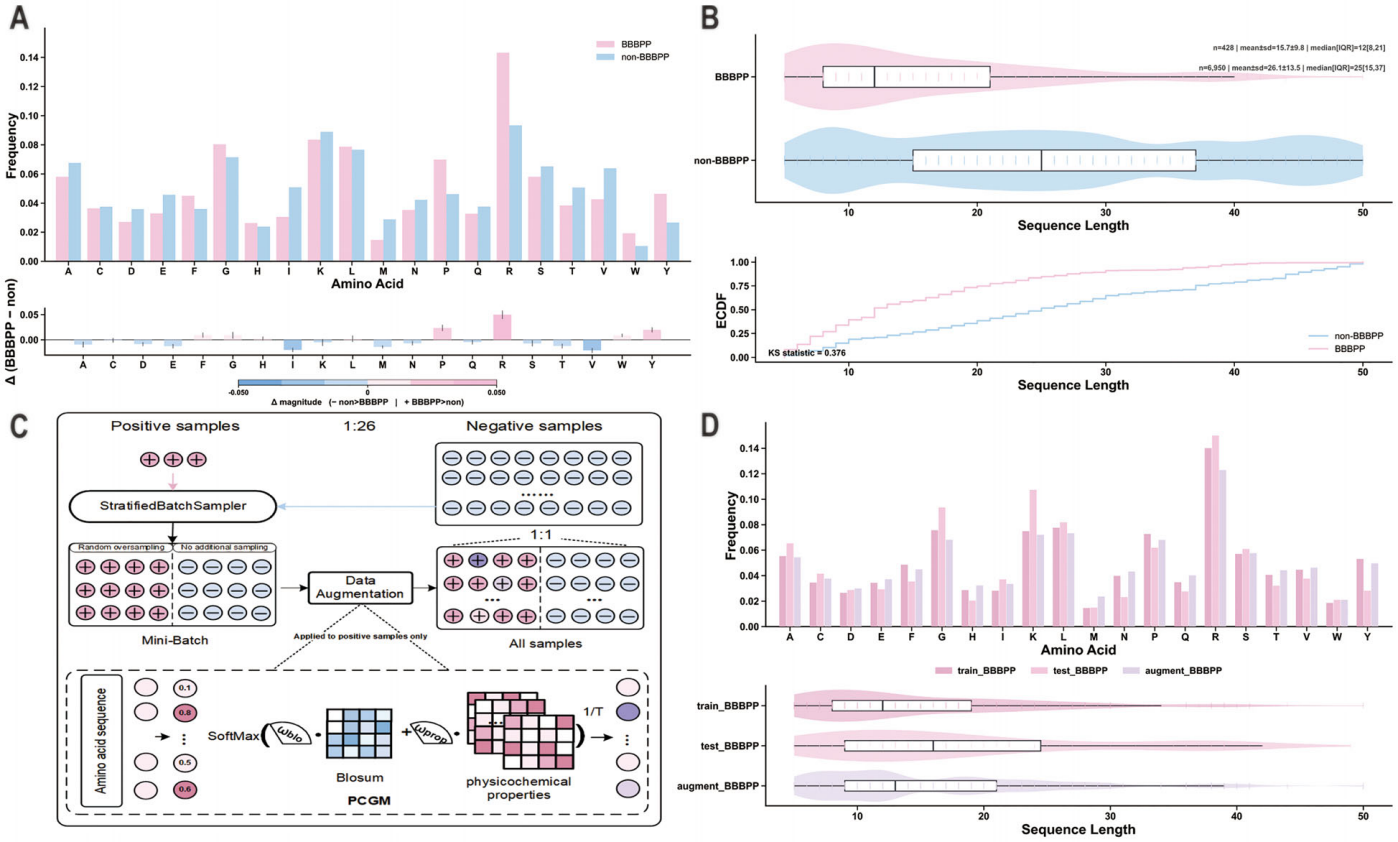
Statistical characterization of the BBBPP dataset and overview of the PCGM data‐augmentation framework. (A) Amino‐acid frequency profiles of BBBPP (pink) and non‐BBBPP (blue) sequences in the combined training and test sets. Bars show the marginal frequency of each of the 20 canonical residues; the lower panel reports the difference in frequency (Δ = BBBPP − non‐BBBPP) with 95% confidence intervals and a diverging color scale, highlighting residues that are comparatively enriched or depleted in BBBPPs. (B) Sequence‐length distributions for BBBPP and non‐BBBPP peptides. Horizontal violin and box plots summarize central tendency and dispersion for each class, and the lower panel shows ECDFs; the Kolmogorov–Smirnov (KS) statistic quantifies the global difference between the two length distributions. (C) Schematic of the sampling and PCGM (physicochemical‐guided mutagenesis) augmentation pipeline used during training. Under the original extreme imbalance (about 1:20.8 BBBPP:non‐BBBPP), a stratified mini‐batch sampler performs random oversampling of BBBPPs to construct approximately balanced batches (close to 1:1). Data augmentation is applied only to oversampled BBBPP sequences. At selected positions, PCGM proposes amino‐acid substitutions according to a residue‐specific probability kernel that linearly combines BLOSUM62 scores with a physicochemical‐similarity kernel derived from a nine‐dimensional property vector. The fused scores are normalized row‐wise with a temperature‐controlled softmax to yield substitution probabilities *P*(*i* → *j*), with self‐substitutions disallowed, thereby favoring conservative and semi‐conservative, physicochemically plausible mutations. (D) Evaluation of the augmented BBBPP set. Amino‐acid composition (top) and sequence‐length distributions (bottom; horizontal violins with embedded box plots and rug plots) are shown for BBBPPs from the training set, the independent test set, and the PCGM‐augmented set. The close agreement between augmented and real BBBPP distributions indicates that PCGM increases positive‐class diversity while preserving low‐order sequence statistics.

To contextualize the learning problem, we first examined global sequence statistics of BBBPP and non‐BBBPP peptides in the combined training and test sets (Figure 2A,B). At the level of amino‐acid composition, the two classes show broadly similar overall profiles but display systematic differences at selected residues (Figure 2A). BBBPPs exhibit modest enrichment of basic and polar residues together with slightly elevated frequencies of certain aromatic and flexible residues, whereas non‐BBBPPs are comparatively enriched in several aliphatic and polar amino acids. The magnitude of these shifts is generally small, with absolute differences in marginal frequency typically below 0.05, indicating that BBBPPs do not form a narrowly defined motif class but instead occupy a compositional regime that is only weakly biased relative to non‐BBBPPs.

Sequence length displays a more pronounced bias between classes (Figure 2B). BBBPPs are generally shorter and less dispersed than non‐BBBPPs, as reflected by lower medians and narrower interquartile ranges in the horizontal violin and box plots. The empirical cumulative distribution functions further emphasize this shift: the BBBPP empirical cumulative distribution functions (ECDF) [12] rise more steeply at shorter lengths, whereas the non‐BBBPP curve accumulates more gradually and extends to longer peptides. The Kolmogorov–Smirnov statistic [13] (KS = 0.376) confirms a substantial difference between the two length distributions. Nevertheless, the curves overlap across a wide interval, implying that length alone cannot fully explain BBBPP status and that more nuanced sequence‐ and structure‐level determinants must be captured by the model.

The sampling and augmentation strategy used during training is summarized in Figure 2C and described in detail in the Materials and Methods section. Briefly, the original dataset is extremely imbalanced, with BBBPPs comprising only a small fraction of all peptides (about 1:20.8). To mitigate this, a stratified mini‐batch sampler performs random oversampling of BBBPPs to construct approximately balanced batches with a ratio close to 1:1. PCGM is then applied exclusively to oversampled BBBPP sequences. At selected positions, substitutions are sampled from a residue‐specific kernel that linearly combines BLOSUM62 scores with a physicochemical‐similarity kernel derived from a nine‐dimensional property vector. The fused scores are normalized row‐wise using a temperature‐controlled SoftMax to yield substitution probabilities *P*(*i* → *j*), with self‐substitutions disallowed. This design biases augmentation toward conservative and semi‐conservative, physicochemically plausible mutations while preserving the global sequence context.

We next assessed whether PCGM preserves the low‐order statistics characterized above (Figure 2D). Focusing on BBBPP sequences only, we compared amino‐acid composition and length distributions for training BBBPPs, independent‐test BBBPPs, and PCGM‐augmented BBBPPs. Across all 20 residues, the augmented set closely tracks the profiles of the real BBBPPs, with only minor fluctuations at individual amino acids and no systematic drift toward the non‐BBBPP composition. Likewise, the length distributions of augmented peptides are well aligned with those of the original BBBPPs: medians and interquartile ranges are similar, and only a modest extension of the upper tail is observed. Together, these analyses indicate that PCGM increases the diversity and effective sample size of the positive class while maintaining global compositional and length characteristics that are consistent with experimentally observed BBBPPs, thereby providing a statistically faithful basis for downstream model training.

### 2.2 Conformity of the Augmented Data to Biochemical Regularities

**FIGURE 3:**
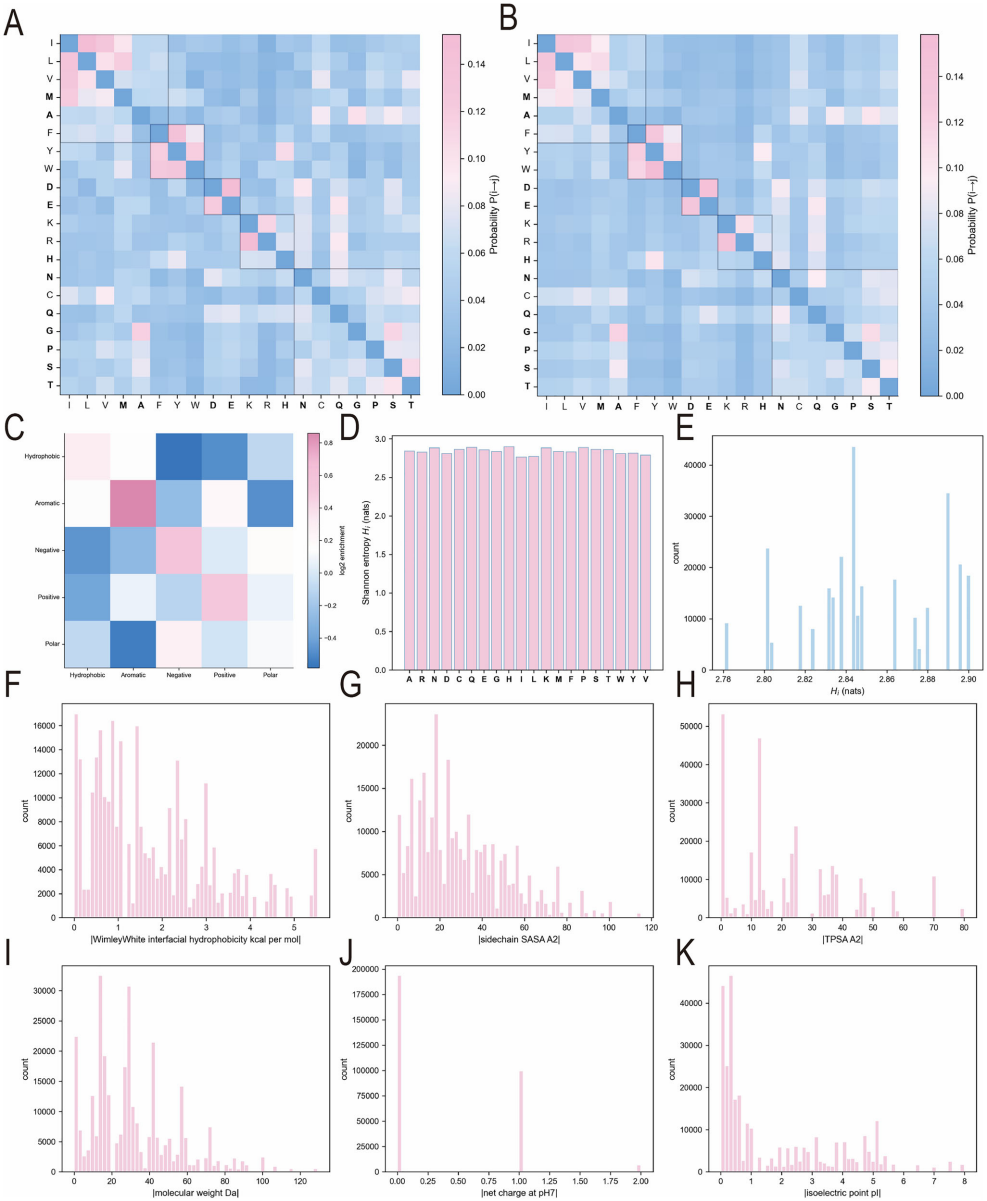
Characterization of PCGM Substitution Patterns and Property Shifts. (A) PCGM soft‐kernel substitution probabilities *P*_PCGM_(*i*  →  *j*). (B) Empirical substitution probabilities *P*_emp_(*i*  →  *j*) from the augmentation logs. Residue‐class blocks (hydrophobic, aromatic, negative, positive, polar) are outlined; bold tick labels denote non‐conservative residues (BLOSUM‐based criterion). (C) Class‐level substitution propensities (row: source class *g*, column: target class *h*). (D) Per‐row Shannon entropy *H_i_* (nats); dashed line marks ln 20. (E) Distribution of event‐level entropies. (F–K) Absolute per‐event changes ∣Δ∣in six properties (interfacial hydrophobicity, side‐chain SASA, TPSA, molecular weight, net charge at pH 7, and pI). Together, these panels quantify substitution structure and property shifts under PCGM without relying on a single metric.

To evaluate whether augmentation preserves realistic sequence chemistry, we contrasted the intended PCGM substitution kernel with the realized substitutions captured in the logs and then quantified diversity and physicochemical drift (Figure 3A–K). The PCGM soft kernel *P*_PCGM_(*i*  →  *j*) is constructed by blending BLOSUM62 with a physicochemical‐similarity prior and applying a row‐wise SoftMax with temperature τ. Empirical probabilities *P*_emp_(*i*  →  *j*) were estimated from all generated events. For interpretability, residues were clustered and five broad classes were outlined—hydrophobic (I, L, V, M, F, A), aromatic (F, W, Y), negative (D, E), positive (K, R, H), and polar/other (S, T, N, Q, C, G, P); bold tick labels mark “non‐conservative” sources with low exchangeability under a BLOSUM‐based criterion. At a glance, *P*_PCGM_ (Figure 3A) and *P*_emp_ (Figure 3B) share the same large‐scale geometry: mass concentrates within chemically proximate blocks (e.g., D↔E, K↔R, I↔L↔V) and is depleted for cross‐class jumps (e.g., charged→aromatic). Importantly, heterogeneity within blocks is expected and informative, not contradictory to “within‐class substitution.” Several sources—P (ring‐constrained backbone), G (high backbone flexibility), C (disulfide chemistry), W/Y (bulky aromatics with packing/H‐bond roles), and H (pH‐dependent protonation)—show attenuated outgoing mass even to nominal peers. In other words, letters that are non‐conservative globally also remain comparatively restrained within their class, reflecting genuine structural/electronic constraints rather than drift of the kernel.

To summarize behavior beyond individual cells, we aggregated *P*_emp_ over classes to obtain group‐to‐group propensities *g*  →  *h* (Figure 3C) and expressed them as log_2_ enrichment over expectations from column mass. The diagonal is enriched (hydrophobic→hydrophobic; positive→positive), while chemically distant transitions are depleted (e.g., charged→aromatic), confirming conservative substitution at the class level.

Diversity was quantified using row entropies [14], reported in nats (max  =  ln20; Figure 3D).

(1) $$H_{i}=-\sum_{j}P_{emp}\left(i\to j\right)InP_{emp}\left(i\to j\right)$$

Values cluster well below the uniform limit yet far from zero, indicating exploration of multiple plausible targets per source residue without collapsing to a single option or drifting toward indiscriminate randomness. When event‐level entropies were logged, their histogram (Figure 3E) peaks at intermediate values with a long, low‐mass tail—again consistent with selective, kernel‐guided exploration.

Event‐wise physicochemical drift was assessed via the absolute change ∣Δ∣ in six properties spanning amphiphilicity, size, and charge (Figure 3F–K): Wimley–White interfacial hydrophobicity, side‐chain SASA, TPSA, molecular weight, net charge at pH 7, and isoelectric point (pI). All six distributions are sharply concentrated near zero with low‐frequency long tails, showing that most substitutions are local in chemical space while admitting occasional larger hops that broaden coverage. Two diagnostic patterns stand out: (i) net‐charge changes (Figure 3J) are strongly centered at 0 with sparse ±1 spikes, indicating charge preservation in the vast majority of events; and (ii) pI shifts (Figure 3K) are heavily right‐skewed toward small increments, suggesting near‐invariance of global electrostatics. Hydrophobicity and SASA (Figure 3F–G) show light‐tailed spreads consistent with conservative side‐chain swaps within size/hydropathy bands.

Taken together—structural agreement between *P*_PCGM_ and *P*_emp_ (Figure 3A,B), diagonal class‐level enrichment (Figure 3C), intermediate entropy consistent with controlled exploration (Figure 3D,E), and small per‐event property shifts with rare larger excursions (Figure 3F–K)—the augmented data exhibit controlled diversity aligned with biochemical regularities, including appropriate restraint for globally non‐conservative sources (P, G, C, W/Y, H). This profile reduces the risk of augmentation‐induced distribution shift during training while still expanding sequence coverage in a chemically coherent manner.

### 2.3 Evaluation of the Proposed Model and Its Predictive Performance Against Prior Baselines

**FIGURE 4:**
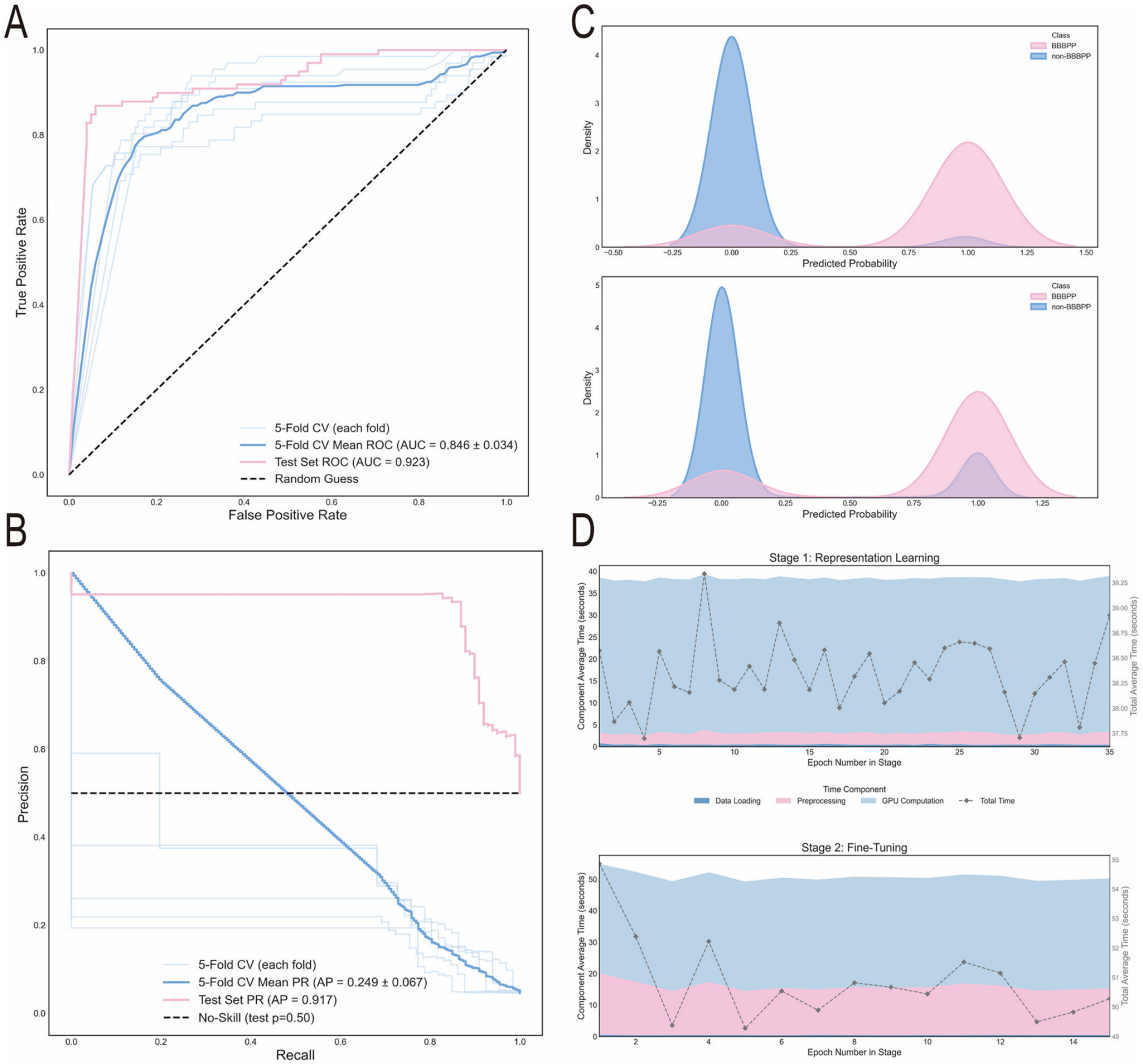
Performance and computational profile of the proposed model. (A) ROC curves: per‐fold CV traces (light blue), mean ROC across folds (AUC =  0.846  ±  0.034), and the independent test set (AUC   0.923). The 45° dashed line indicates random guessing. (B) Precision–recall curves: per‐fold CV traces (light), CV mean AP =  0.249  ±  0.067, and test AP =  0.917. The dashed black line marks the no‐skill baseline equal to the positive‐class prevalence in the test set (∼0.50). All ROC/PR metrics are threshold‐agnostic and computed from out‐of‐fold predictions for CV and from the untouched test set. (C) Kernel‐density estimates of predicted probability *p*(BBBPP). Top, independent test set; bottom, pooled out‐of‐fold predictions from fivefold CV. (D) Per‐epoch training‐time decomposition, averaged across repeated runs (here *R*  =  3). Data loading = time waiting for the next batch from the DataLoader (disk I/O, worker → main process queueing/prefetch). Preprocessing = CPU‐side transforms after a batch arrives and before GPU launch (tokenization, PCGM substitutions, padding/masking, feature assembly/graph construction). GPU computation = device‐side work (host→device copies, forward pass, loss, backward, optimizer/scheduler, and any gradient synchronization). Total time (gray dashed, right *y*‐axis) = data loading + preprocessing + GPU computation. Because prefetching can overlap data loading with compute, this sum upper‐bounds true wall‐clock time (epoch wall time is reported in Methods). Stage‐1 corresponds to representation learning; Stage‐2 to fine‐tuning.

We first characterize model behavior on held‐out data and quantify the computational profile (Figure 4). In ROC space (Figure 4A), the independent test set yields AUROC = 0.923, indicating strong threshold‐agnostic discrimination. Across five folds, the mean AUROC is 0.846 ± 0.034; fold‐level curves lie uniformly above the diagonal with limited dispersion, consistent with stable generalization under resampling rather than split‐specific effects. Precision–recall analysis (Figure 4B) situates ranking performance relative to prevalence: the test set attains AP = 0.917, whereas the five‐fold mean is 0.249 ± 0.067. Dashed horizontal lines mark the no‐skill baselines (equal to the evaluation prevalence), clarifying that gains in AP exceed what prevalence alone would afford. The test‐set PR curve sustains high precision across a broad recall range—appropriate for screening scenarios where false positives are costly—while cross‐validation curves exhibit a consistent, albeit more conservative, profile. These threshold‐agnostic findings accord with the score distributions in Figure 4C: class‐conditional kernel density estimates of p(BBBPP) display well‐separated, mildly bimodal densities for both the independent test set and pooled out‐of‐fold predictions, with limited overlap near the decision boundary. This distributional evidence suggests that the classifier establishes a stable margin between BBB‐penetrating and non‐BBB peptides rather than exploiting idiosyncrasies of any particular split. Finally, Figure 4D decomposes per‐epoch training time into data loading (disk I/O and DataLoader queueing), preprocessing (CPU‐side tokenization, PCGM substitutions, padding/masking, and feature/graph assembly), and GPU computation (host‐to‐device transfers; forward, loss, and backward passes; optimizer/scheduler updates; gradient synchronization). The gray dashed curve (right axis) is the component sum; because prefetching overlaps I/O with compute, it serves as an upper bound on the true wall‐clock time per epoch. Stage 1 (representation learning) is compute‐dominant with non‐trivial preprocessing, whereas Stage 2 (fine‐tuning) is lighter and less variable—consistent with freezing most backbone layers and amortizing representation shaping upfront. Together, these properties support both predictive robustness and practical training efficiency.

To situate these results relative to prior art, we evaluated representative BBBPP predictors under identical splits and inputs (Table 1). INB^3^P achieves the strongest thresholded performance on the independent test set—sensitivity (Sn) = 0.8283, specificity (Sp) = 0.9495, accuracy (ACC) = 0.8889, and Matthews correlation coefficient (MCC) = 0.7836—and improves upon the strongest baseline (DeepB^3^P) by 6.1% in Sn, 8.1% in Sp, 7.1% in ACC and 114.4% in MCC. These gains are not the result of a recall–specificity trade‐off: both Sn and Sp increase. Because the test set is class‐balanced, balanced accuracy equals ACC for all models, enabling direct comparison without prevalence confounds. Taken together with the threshold‐agnostic evidence in Figure 4A,B and the diagnostic separability in Figure 4C, these comparative results indicate practically meaningful improvements over prior methods rather than artifacts of thresholding or dataset partitioning.

**TABLE 1:** Comparison with prior BBBPP predictors on the independent test set.

Model	Sn	Sp	ACC	MCC	Refs.
B^3^Pred	0.7071	0.6465	0.6768	0.3542	[15]
BBPpred	0.6768	0.6566	0.6667	0.3334	[16]
BBPpredict	0.7677	0.7778	0.7727	0.5455	[17]
DeepB^3^P	0.7677	0.8687	0.8182	0.6396	[2]
INB^3^P(ours)	0.8283	0.9495	0.8889	0.7836	

### 2.4 Contribution of Model Components and Loss Functions

**FIGURE 5:**
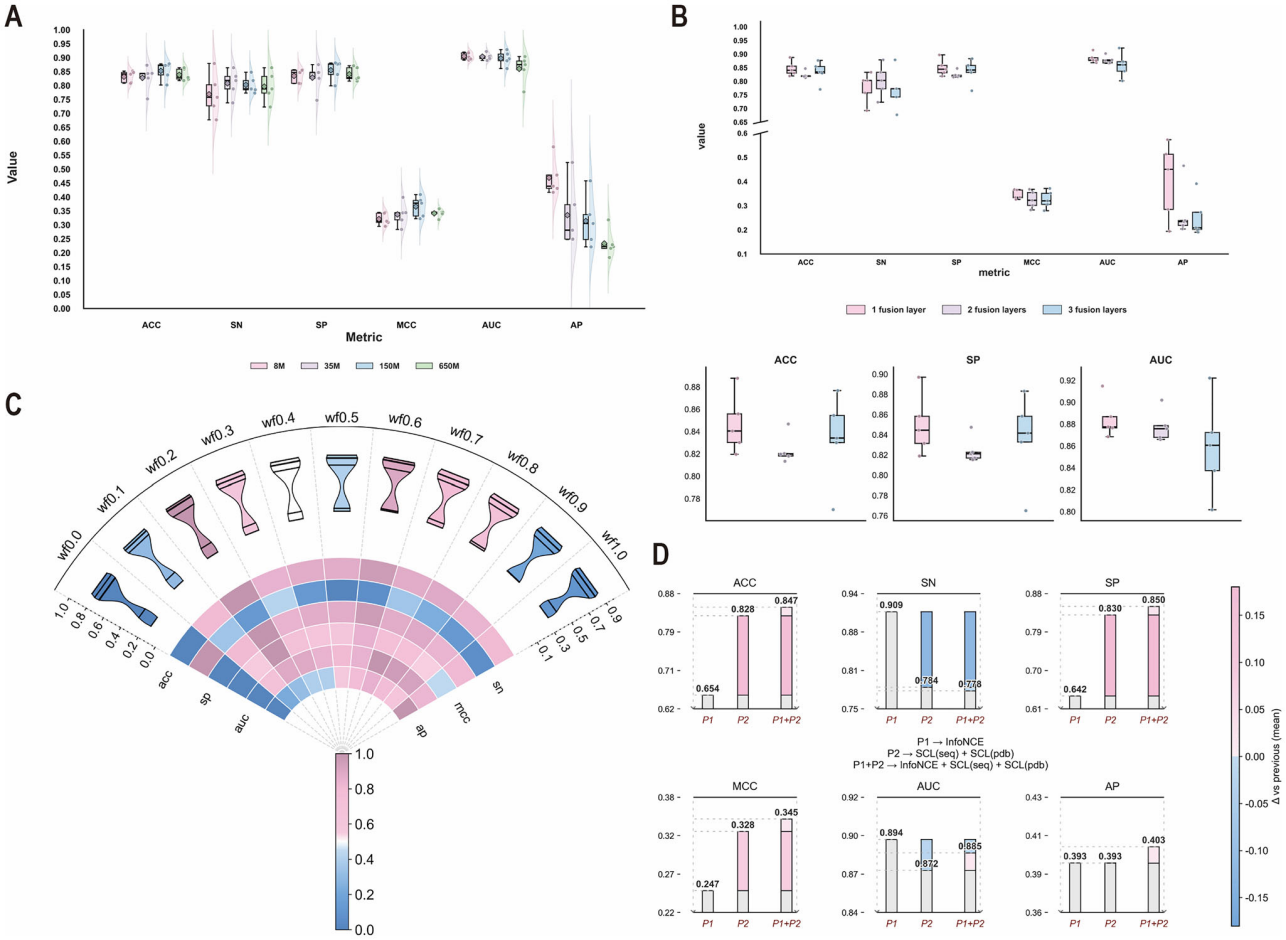
Contribution of architectural components and loss functions to INB^3^P performance. (A) ESM‐2 backbone selection. Effect of using different ESM‐2 variants (8, 35, 150, and 650 M) as the sequence encoder. Box–violin plots summarize fivefold cross‐validation performance across six metrics (ACC; Sn; Sp; MCC; AUC and AP). Larger backbones generally improve MCC, AUC, and AP, with performance gains diminishing for the largest models. (B) Depth of the fusion module. Influence of stacking 1, 2, or 3 sequence–structure fusion layers. The upper panel reports the distribution of all metrics over fivefolds, whereas the lower panel focuses on ACC, Sp, and AUC. Two fusion layers provide a favourable balance between discrimination and stability, while further increasing depth yields at most modest additional benefit and slightly higher variability. (C) Tuning the classification‐loss weights. Fan‐shaped radial violin plot depicting fivefold performance as a function of the focal‐loss weight *w*_focal_in the composite classification loss *L*_cls_  =  *w*_focal_ *L*_focal_  +  (1  −  *w*_focal_)*L*_Stable‐MCC_. Each spoke corresponds to one setting (wf0.0–wf1.0); outer violins show the distribution of each metric, and concentric coloured bands encode normalized performance. Intermediate values of *w*_focal_yield the most balanced overall profile across ACC, Sn, Sp, MCC, AUC, and AP, whereas extreme weights favour only subsets of metrics. (D) Loss‐function ablation and two‐stage curriculum. Bar plots comparing fivefold performance for three training objectives: P1, InfoNCE only; P2, supervised contrastive losses on sequence and structure embeddings only (SCL(seq)+SCL(pdb)); and P1+P2, the combined objective adopted in INB^3^P. Bars report mean metric values, and shading reflects the change relative to the worst configuration. The combined InfoNCE+SCL objective achieves the highest overall discrimination (MCC, AUC, and AP) while maintaining a balanced sensitivity–specificity trade‐off.

To elucidate how individual design choices affect INB^3^P, we performed a series of ablation experiments on the sequence encoder, fusion depth, classification‐loss weighting, and contrastive objectives. Cross‐validated summary statistics (mean ± s.d. over five folds) are reported in Tables 2, 3, 4, 5 and visualised in Figure 5; the corresponding fold‐wise values are provided in Tables S1–S4, respectively. In addition, to further clarify the contribution of each modality and the effectiveness of the fusion mechanism, we conducted supplementary architecture‐level ablations in two aspects: first, modality‐level comparisons among sequence‐only, structure‐only, and full multimodal variants; second, fusion‐level comparisons among concatenation‐based fusion, cross‐attention‐based fusion, and the full model. The corresponding results are reported in Note S1.

**TABLE 2:** Ablation study on the scaling of the ESM‐2 sequence encoder.

ESM‐2 version	AUC	AP	MCC	ACC	Sn	Sp	Time^*^ (s/epoch)
8 M	0.905 ± 0.013	0.469 ± 0.066	0.321 ± 0.022	0.832 ± 0.021	0.769 ± 0.077	0.835 ± 0.025	11.958 ± 0.393
35 M	0.904 ± 0.012	0.335 ± 0.117	0.338 ± 0.042	0.828 ± 0.046	0.808 ± 0.048	0.828 ± 0.049	13.900 ± 0.550
150 M	0.898 ± 0.026	0.314 ± 0.093	0.366 ± 0.037	0.853 ± 0.033	0.802 ± 0.030	0.855 ± 0.036	21.805 ± 0.442
650 M	0.860 ± 0.050	0.234 ± 0.050	0.342 ± 0.014	0.839 ± 0.021	0.796 ± 0.055	0.841 ± 0.025	43.619 ± 0.440

* Time denotes the per‐epoch runtime (seconds), jointly accounting for Stage‐1 (35 epochs) and Stage‐2 (15 epochs). For each fold, we first sum the per‐epoch means of modules A and B and approximate the combined SD as $s_{\mathrm{tot}}=\sqrt{s_{A}^{2}+s_{B}^{2}}$; we then compute a pooled summary weighted by the stage epoch counts (35 and 15), and finally report the fivefold mean ± pooled SD.

**TABLE 3:** Ablation analysis on the depth of the cross‐modal fusion module.

Fusion layer	AUC	AP	MCC	ACC	Sn	Sp	Time^*^ (s/epoch)
**1**	0.847 ± 0.038	0.250 ± 0.075	0.317 ± 0.022	0.819 ± 0.033	0.790 ± 0.057	0.820 ± 0.037	32.896 ± 0.459
**2**	0.861 ± 0.041	0.252 ± 0.083	0.349 ± 0.033	0.849 ± 0.007	0.784 ± 0.058	0.852 ± 0.006	34.437 ± 0.463
**3**	0.646 ± 0.305	0.192 ± 0.188	0.082 ± 0.184	0.220 ± 0.389	0.932 ± 0.151	0.186 ± 0.415	35.907 ± 0.454
**4**	0.659 ± 0.176	0.210 ± 0.238	0.094 ± 0.207	0.221 ± 0.391	0.948 ± 0.115	0.186 ± 0.415	37.469 ± 0.459

* Time denotes the per‐epoch runtime (seconds), jointly accounting for Stage‐1 (35 epochs) and Stage‐2 (15 epochs). For each fold, we first sum the per‐epoch means of modules A and B and approximate the combined SD as $s_{\mathrm{tot}}=\sqrt{s_{A}^{2}+s_{B}^{2}}$; we then compute a pooled summary weighted by the stage epoch counts (35 and 15), and finally report the fivefold mean ± pooled SD.

**TABLE 4:** Ablation study on contrastive learning objectives during the representation learning stage.

Loss function	AUC	AP	MCC	ACC	Sn	Sp
InfoNCE	0.906 ± 0.012	0.419 ± 0.047	0.319 ± 0.015	0.802 ± 0.022	0.839 ± 0.062	0.801 ± 0.026
SCL(seq) + SCL(pdb)	0.867 ± 0.031	0.289 ± 0.109	0.354 ± 0.029	0.852 ± 0.010	0.787 ± 0.059	0.855 ± 0.011
InfoNCE + SCL(seq) + SCL(pdb)	0.847 ± 0.038	0.249 ± 0.075	0.317 ± 0.022	0.819 ± 0.033	0.790 ± 0.057	0.820 ± 0.037

**TABLE 5:** Tuning the weight balance between Focal Loss and Stable‐MCC for imbalance‐robust classification.

short_key^*^	Sn	Sp	MCC	ACC	AUC	AP
wf0.20	0.778 ± 0.045	0.860 ± 0.008	0.356 ± 0.016	0.857 ± 0.006	0.883 ± 0.024	0.237 ± 0.038
wf0.60	0.775 ± 0.051	0.855 ± 0.008	0.348 ± 0.035	0.851 ± 0.009	0.878 ± 0.026	0.362 ± 0.179
wf0.70	0.790 ± 0.049	0.844 ± 0.012	0.341 ± 0.021	0.841 ± 0.011	0.895 ± 0.006	0.387 ± 0.132
wf0.80	0.784 ± 0.039	0.845 ± 0.015	0.340 ± 0.014	0.842 ± 0.013	0.890 ± 0.021	0.364 ± 0.148
wf0.30	0.793 ± 0.029	0.840 ± 0.030	0.340 ± 0.025	0.837 ± 0.028	0.883 ± 0.025	0.260 ± 0.121
wf0.40	0.778 ± 0.053	0.844 ± 0.012	0.336 ± 0.015	0.841 ± 0.010	0.877 ± 0.013	0.260 ± 0.040
wf0.50	0.772 ± 0.062	0.844 ± 0.022	0.334 ± 0.038	0.841 ± 0.021	0.872 ± 0.041	0.305 ± 0.150
wf0.10	0.790 ± 0.080	0.834 ± 0.018	0.330 ± 0.034	0.832 ± 0.016	0.861 ± 0.047	0.211 ± 0.037
wf0.90	0.778 ± 0.061	0.839 ± 0.017	0.330 ± 0.025	0.836 ± 0.015	0.887 ± 0.009	0.326 ± 0.117
wf1.00	0.775 ± 0.039	0.835 ± 0.016	0.324 ± 0.025	0.832 ± 0.015	0.875 ± 0.026	0.427 ± 0.126

* short_key is a shorthand identifier for each loss configuration. The label “wfX.XX” encodes the focal‐loss weight *w*_focal_ =  *X*.*XX*(rounded to two decimal places), with the complementary MCC‐loss weight *w*_MCC_ =  1 − *w*_focal_; all runs use the same focal focusing parameter γ_focal_ =  2and identical training settings. Rows are ordered from top to bottom by decreasing mean MCC over five‐fold cross‐validation, using mean AUC as a secondary ranking criterion when mean MCC values are similar.

#### 2.4.1 ESM‐2 Backbone Size

We first assessed the impact of the sequence encoder by replacing the backbone with four ESM‐2 variants (8, 35, 150, and 650 M parameters; Figure 5A, Table 2). With the 8, 35, and 150 M models, AUROC and MCC were broadly comparable (AUC ≈ 0.90; MCC = 0.321–0.366), with the 150 M variant achieving the highest MCC (0.366 ± 0.037), accuracy (0.853 ± 0.033), and specificity (0.855 ± 0.036). The largest 650 M model exhibited slightly reduced cross‐validated metrics (AUC = 0.860 ± 0.050, MCC = 0.342 ± 0.014, ACC = 0.839 ± 0.021), although the differences were modest given the variability across folds. At the same time, the 650 M backbone yields the most expressive, context‐rich residue representations and has been shown to encode fine‐grained structural and evolutionary information that is particularly beneficial for contrastive and transfer learning. Considering the overall similarity in performance among the 8–150 M variants, and the potential downstream advantages of a more informative representation space, we selected ESM‐2 650 M as the default backbone for INB^3^P, accepting the increased per‐epoch runtime (43.62 ± 0.44 s vs. 11.96–21.81 s for the smaller models) in exchange for a higher‐capacity feature extractor that can be reused throughout our pipeline.

#### 2.4.2 Depth of the Fusion Module

We next examined the number of sequence–structure fusion layers required to exploit complementary modalities (Figure 5B, Table 3). A single fusion layer already delivered strong performance (AUC = 0.847 ± 0.038, MCC = 0.317 ± 0.022, ACC = 0.819 ± 0.033) with a balanced sensitivity–specificity profile (Sn = 0.790 ± 0.057, Sp = 0.820 ± 0.037) and moderate computational cost (32.90 ± 0.46 s per epoch). Introducing a second fusion layer further increased AUC and MCC (0.861 ± 0.041 and 0.349 ± 0.033, respectively) and improved ACC and Sp, at the price of only a slight increase in runtime (34.44 ± 0.46 s) and a small decrease in Sn. However, deeper configurations with three or four fusion layers behaved pathologically: they produced very high sensitivities (>0.93) but extremely low specificities (∼0.19), leading to poor MCC (0.082–0.094) and ACC (∼0.22) with large variances. These degenerate solutions indicate overfitting and a strong bias toward predicting the positive class when the fusion module is overly deep. Given that a single fusion layer already affords robust performance with a simpler architecture, lower variance, and reduced interpretability burden, we adopted one fusion layer in the final INB^3^P model and regard the two‐layer configuration as an upper bound on potential gains.

#### 2.4.3 Weighting of Focal and Stable‐MCC Classification Losses

To optimize the classification objective under severe class imbalance, we tuned the relative contributions of focal loss and the stable‐mcc loss in the composite term.

(2) $$L_{\mathrm{cls}}=w_{\mathrm{focal}}\;L_{\mathrm{focal}}+\left(1-w_{\mathrm{focal}}\right)L_{\mathrm{Stable}-\mathrm{MCC}}$$

We scanned *w*_focal_from 0 to 1 in increments of 0.1 (settings wf0.0–wf1.0; Figure 5C, Table 4). Extreme choices were suboptimal: pure Stable‐MCC (wf0.00) and pure focal loss (wf1.00) both resulted in MCC ≈ 0.33 and ACC ≈ 0.83, with either reduced AUC or less balanced sensitivity–specificity trade‐offs. In contrast, the wf0.20 configuration (*w*_focal_  =  0.20,  *w*_MCC_  =   0.80) yielded the highest MCC (0.356 ± 0.016) and accuracy (0.857 ± 0.006), combined with strong specificity (0.860 ± 0.008) and competitive AUC (0.883 ± 0.024). Larger focal weights (wf0.70–wf1.00) improved rank‐based metrics such as AUC and AP but did not translate into superior MCC or ACC. These observations highlight that heavily weighting the Stable‐MCC term while retaining a modest focal component stabilises the decision boundary at clinically relevant operating points and underscores the effectiveness of our Stable‐MCC formulation for imbalanced BBBPP prediction. Consequently, we use *w*_focal_  =  0.20, *w*_MCC_  =  0.80in all subsequent experiments.

#### 2.4.4 Loss‐Function Ablation and Contrastive Objectives

Finally, we dissected the contribution of contrastive objectives used in Stage‐1 representation learning (Figure 5D, Table 5). Training with InfoNCE alone (P1) achieved the highest AUROC (0.906 ± 0.012) and AP (0.419 ± 0.047) and the largest sensitivity (0.839 ± 0.062), but at the expense of lower ACC (0.802 ± 0.022), MCC (0.319 ± 0.015), and specificity (0.801 ± 0.026). Using only supervised contrastive losses on sequence and structure embeddings (P2; SCL(seq)+SCL(pdb)) produced superior threshold‐dependent metrics (MCC = 0.354 ± 0.029, ACC = 0.852 ± 0.010, Sp = 0.855 ± 0.011) but reduced AUC (0.867 ± 0.031), AP (0.289 ± 0.109), and recall of positive samples. The combined objective P1 + P2, which we adopt in the final INB^3^P configuration, yielded intermediate yet well‐balanced performance across all six metrics (AUC = 0.847 ± 0.038, MCC = 0.317 ± 0.022, ACC = 0.819 ± 0.033), reflecting a compromise between the ranking advantages conferred by InfoNCE and the calibration benefits of supervised contrastive learning. Together with the loss‐weight tuning results, these ablations support our final design choices—ESM‐2 650 M backbone, a single fusion layer, a focal: Stable‐MCC weight of 0.2:0.8, and the combined P1 + P2 objective—as a principled configuration that balances predictive accuracy, robustness, and representational richness.

### 2.5 Interpretability Study and Biological Insights

**FIGURE 6:**
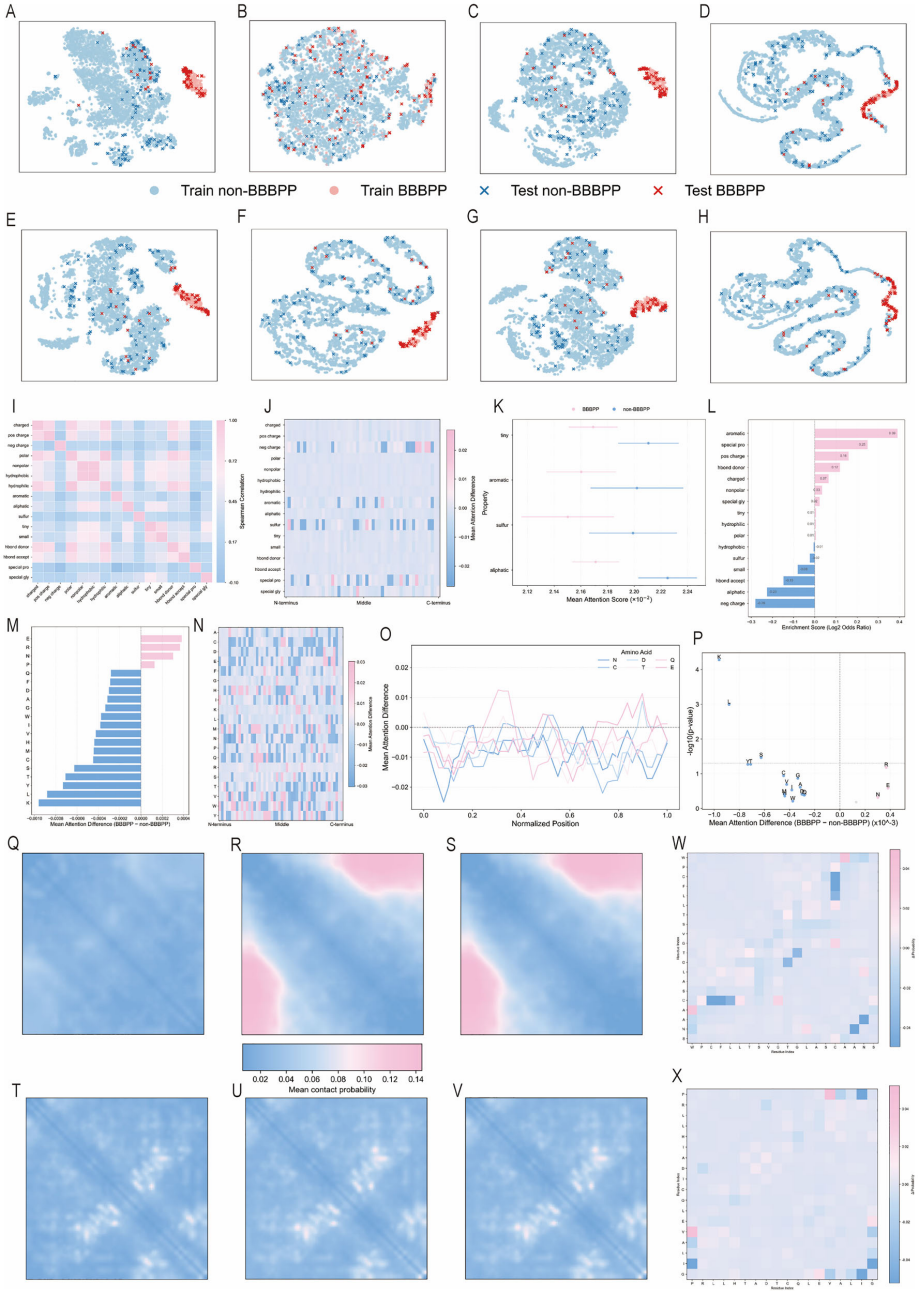
Multi‐level interpretability analyses of INB^3^P across latent spaces, physicochemical properties, amino‐acid usage, and contact maps. (A–H) Two‐dimensional t‐SNE projections of eight intermediate representations extracted from INB^3^P: initial ESM‐2 sequence embeddings, initial graph‐based structural embeddings, post‐fusion sequence and structure encodings, concatenated pre‐MLP fused representations, final post‐MLP fused representations, and the supervised‐contrastive (SCL) sequence and structure projections. Each point corresponds to a peptide (circles, training set; crosses, independent test set), with colors distinguishing BBBPPs from non‐BBBPPs (common legend). These panels allow visual comparison of how sequential encoding, cross‐modal fusion, and contrastive objectives reshape the embedding space. (I–L) Property‐level analysis based on sequence‐to‐property attention scores from the final model. (I) Log_2_ odds‐ratio enrichment of physicochemical properties among the most highly attended residues in BBBPPs, quantifying which properties are preferentially emphasized by the model. (J) Spearman correlation heatmap of property‐wise mean attention vectors across BBBPP sequences, highlighting co‐attended property clusters. (K) Mean attention (±95% confidence intervals) for the four properties with the largest absolute BBBPP–non‐BBBPP differences, plotted separately for BBBPP and non‐BBBPP peptides. (L) Heatmap of BBBPP–non‐BBBPP attention differences for each property along the normalized sequence axis (N‐terminus to C‐terminus), summarizing how property focus varies across sequence position and class. (M–P) Dataset‐level amino‐acid attention analysis. A combined dashboard (bar plot and heatmap) summarizes the mean attention difference (BBBPP − non‐BBBPP) for each residue type and its positional dependence along the sequence. Additional panels show (i) position‐resolved attention differences for individual amino acids (N‐terminus to C‐terminus), (ii) smoothed positional profiles for residues with the largest absolute attention shifts, and (iii) a volcano‐like plot relating mean attention differences to −log_10_ *p*‐values, with residues (or short motifs) passing joint effect‐size and significance thresholds annotated. Together, these views characterize which amino acids and positions are most discriminative in the learned attention patterns. (Q–V) Classwise mean contact maps derived from the ESM‐based contact head across training stages. Pretrained, Stage‐1, and Stage‐2 contact probabilities are averaged separately over BBBPPs (Q–S) and non‐BBBPPs (T–V), yielding a 2 × 3 panel of classwise mean contact maps. These panels illustrate how cross‐modal alignment (Stage 1) and subsequent fine‐tuning (Stage 2) systematically reshape the effective contact patterns in a class‐dependent manner. (W–X) Δ‐contact maps, defined as Stage‐2 minus Stage‐1 contact probabilities, for the two peptides exhibiting the largest mean long‐range (|i − j| above a minimum gap) contact changes. Warm and cool colors indicate increases and decreases in predicted contact probability, respectively. These examples demonstrate that Stage‐2 fine‐tuning induces substantial, localized adjustments to long‐range contact structure rather than leaving the pretrained contact prior unchanged.

**FIGURE 7:**
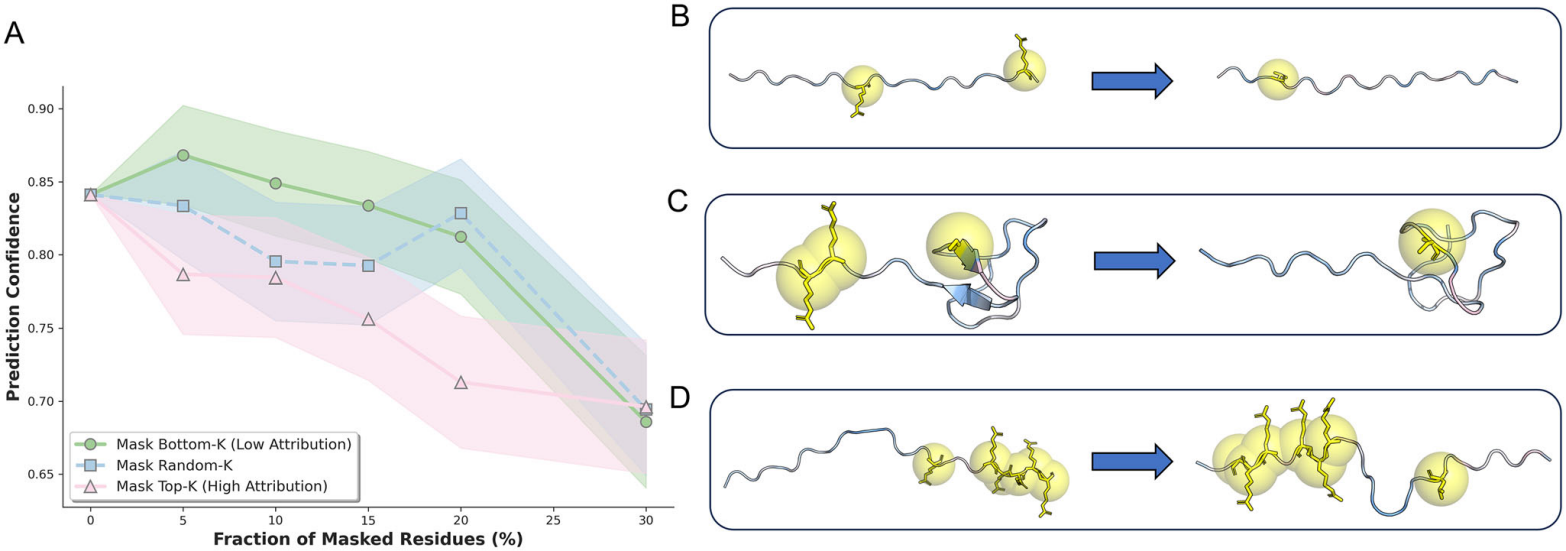
Perturbation‐based faithfulness analysis of INB^3^P and representative structural responses to attribution‐guided residue masking. (A) In silico alanine‐scanning faithfulness curves showing the mean prediction confidence as an increasing fraction of residues is perturbed, comparing masking of the top‐K highest‐attribution residues, bottom‐K lowest‐attribution residues, and randomly selected residues. Shaded regions indicate variability across samples. A stronger confidence decrease under top‐K masking suggests that residues assigned high attribution by the model contribute more strongly to the final prediction than low‐attribution or randomly chosen positions within the current perturbation setting. (B–D) Three representative peptides illustrating structural responses to attribution‐guided perturbation. Residues with high attribution are highlighted in yellow before masking/substitution, and the corresponding post‐perturbation structures are shown on the right. These examples show that perturbing a small number of highly attributed positions can induce detectable local or long‐range conformational rearrangements in the ESMFold‐derived structural representations, consistent with the elevated model sensitivity associated with the identified regions. The blue arrows indicate the transition from the original to the perturbed state.

To clarify how architectural components and loss functions shape what INB^3^P learns about BBBPPs, we performed a multi‐level interpretability analysis spanning latent geometry, physicochemical properties, residue usage, and contact patterns (Figure 6), and further extended this framework with a perturbation‐based faithfulness analysis [18] to assess whether highly attributed residues are genuinely important to the model's predictions. (Figure 7) These analyses are based on embeddings and attention scores, contact‐pattern shifts, and attribution‐guided perturbation responses extracted from trained models, using held‐out data where applicable, and are therefore complementary to the supervised metrics described above. While descriptive rather than strictly causal, they provide convergent computational evidence for the biological relevance of the learned representations. These patterns can be directly compared with the physicochemical and positional design principles that have been reported for BBB‐penetrating peptides.

We first examined the organization of the learned latent space using t‐SNE [19] projections of eight intermediate representations (Figure 6A–H). The raw ESM‐2 sequence and initial graph‐based structural embeddings already show mild separation between BBBPPs and non‐BBBPPs, but with substantial overlap and no clear boundary. After cross‐modal encoding and fusion, the sequence and structure representations become progressively more segregated by class, and the pre‐ and post‐MLP fused embeddings exhibit a clear margin between BBBPP and non‐BBBPP clusters while maintaining strong overlap between training and independent‐test points. The supervised‐contrastive projection heads for sequence and structure show the most pronounced class separation, with BBBPPs forming compact, coherent clusters and non‐BBBPPs occupying complementary regions in the joint manifold. These trends indicate that the combination of cross‐modal fusion, InfoNCE, and supervised contrastive losses sculpts a representation space in which BBBPPs and non‐BBBPPs are geometrically disentangled without obvious split‐specific artefacts.

We then compared the model's attention over physicochemical properties with established BBBPP design rules. Meta‐analyses of brain‐penetrant shuttles indicate that successful BBBPPs tend to be modestly cationic, moderately hydrophobic, and amphipathic, with a balanced distribution of charge and hydrophobicity rather than extreme values of either property [20, 21]. They typically occupy an “intermediate” chemical space between classical cell‐penetrating peptides (CPPs) and non‐penetrant peptides, with lower charge density, reduced bulk aromaticity, and improved stability [9, 22]. Using a predefined mapping from amino acids to property indicators, we aggregated last‐layer sequence‐to‐property attention scores and computed enrichment among the most highly attended residues (Figure 6I–L). Positively charged, polar, and “special” categories (including Lys/Arg/His, Pro, and Gly) are consistently enriched in the high‐attention set for BBBPPs, whereas purely hydrophobic and strongly negative properties are comparatively de‐emphasized [23]. Property–property correlation maps reveal co‐attended groups that couple cationicity, hydrogen‐bond donation, and polarity—features that are mechanistically consistent with adsorptive‐ and receptor‐mediated transcytosis, where cationic and polar faces of an amphipathic peptide engage anionic headgroups or protein receptors at the endothelial surface [21].

Importantly, these property preferences are not uniform along the sequence. Mean attention profiles with confidence intervals and property‐by‐position heatmaps show that the largest BBBPP–non‐BBBPP differences arise in specific positional bands along the normalized sequence axis (e.g., analogous to CPPs, where uptake is sensitive to the position and clustering of cationic residues and to primary amphipathic domain organization [24, 25]). BBBPPs display elevated attention to cationic and polar properties in defined N‐terminal or central regions, whereas non‐BBBPPs exhibit flatter or differently patterned profiles. This positional structuring accords with the notion that BBBPPs are not simply “cationic” or “hydrophobic” in bulk, but are finely tuned to form amphipathic motifs in which charged and hydrophobic residues are arranged to generate a directional interaction surface for membranes or receptors [26].

At the residue level, we summarized dataset‐wide amino‐acid attention patterns using bar plots, position‐resolved heatmaps, and volcano‐style summaries (Figure 6M–P). Aggregating attention scores per residue type and class reveals systematic differences between BBBPPs and non‐BBBPPs: residues contributing to net positive charge and polarity (e.g., Lys, Arg, His, and certain polar residues) tend to receive higher attention in BBBPPs, whereas strongly hydrophobic or acidic residues are down‐weighted in the same positional context [20, 27]. Residue‐by‐position heatmaps and smoothed profiles highlight that these shifts are spatially organized: cationic and aromatic side chains are preferentially emphasized in discrete segments (e.g., N‐terminal or central windows), consistent with the formation of amphipathic helices or loops upon membrane engagement, while contiguous hydrophobic stretches without compensating charge are relatively disfavored [28, 29]. This pattern is in line with comparative studies showing that BBB‐penetrating peptides exhibit a sparse, balanced distribution of cationic residues embedded within polar and hydrophobic backgrounds, in contrast to CPPs, which often contain long blocks of uninterrupted Arg/Lys and higher overall aromaticity [27]. The volcano analysis confirms that several residue types show statistically robust attention shifts rather than idiosyncratic fluctuations, indicating that these sequence‐level preferences are learned consistently across the cohort.

Although INB^3^P is trained without any explicit structural supervision or auxiliary contact‐map loss, gradients from the classification and contrastive objectives still back‐propagate through the ESM‐2 backbone and its internal contact head [30, 31]. We therefore examined whether fine‐tuning reshapes [32, 33] this pretrained structural prior in a label‐dependent manner (Figure 6Q–X). Class‐wise mean contact maps from the pretrained model show the expected dominance of near‐diagonal (short‐range) contacts but relatively weak, largely indistinguishable long‐range structure for both BBBPPs and non‐BBBPPs. After Stage‐1 representation learning and Stage‐2 fine‐tuning, mean contact maps for BBBPPs exhibit more pronounced and spatially organized long‐range bands, particularly linking distant sequence segments, whereas non‐BBBPP maps display more modest changes and remain dominated by local contacts (Figure 6Q–V). For representative BBBPPs, triptych contact maps (Pretrain, Stage 1, Stage 2) illustrate that long‐range contact patterns become progressively sharper and more coherent after fine‐tuning, consistent with the emergence of stabilized secondary or tertiary motifs that could support receptor engagement or transcytotic transport. Δ‐contact maps (Stage 2 minus Stage 1) for peptides with the largest mean change in long‐range contact probability highlight localized regions where fine‐tuning increases or decreases contact probabilities (Figure 6W–X), suggesting a targeted reweighting of the structural prior rather than a uniform global shift [34, 35, 36].

To move beyond descriptive visualization and assess whether the highlighted residues are genuinely involved in the model's decision process, we further performed a perturbation‐based faithfulness analysis using in silico alanine scanning. Specifically, residues were ranked by attribution score, and increasing fractions of the sequence were perturbed by masking/substitution under three strategies: Top‐K masking of the most highly attributed residues, Bottom‐K masking of the least attributed residues, and Random‐K masking as a neutral control. As shown in Figure 7A, perturbing the highest‐attribution residues consistently caused the largest reduction in prediction confidence across masking ratios, whereas perturbing low‐attribution residues produced a substantially weaker effect, and random masking showed an intermediate behavior. This divergence was most evident at low‐to‐moderate perturbation levels, suggesting that the model's attribution scores are not merely visually salient, but are meaningfully associated with model sensitivity under selective perturbation. In other words, residues prioritized by the explanation map are associated with a disproportionately larger change in the final BBBPP score than equally sized low‐attribution or random perturbations.

To further illustrate this effect, three representative peptides were selected for qualitative visualization of the structural consequences of attribution‐guided perturbation (Figure 7B–D). In these examples, mutation of only a small number of highly attributed residues was sufficient to induce visible local rearrangements or broader long‐range structural changes in the ESMFold‐derived conformational representations. Although these structural shifts should not be interpreted as direct experimental evidence of a transport mechanism, they provide an additional layer of consistency between the model's attribution map and its learned sequence–structure representations. Together, these results strengthen the interpretability claim by showing that the residues emphasized by INB^3^P are not only associated with characteristic physicochemical or structural patterns but also measurably affect the model output when selectively disrupted.

Taken together, these interpretability analyses suggest that the components and loss functions that improve classification performance also shape INB^3^P toward biologically interpretable representations. At the level of latent geometry, the model forms a stable, class‐separable cross‐modal embedding. At the level of sequence chemistry, it emphasizes balanced cationic and polar motifs arranged in position‐dependent patterns compatible with amphipathic organization. At the level of structure, fine‐tuning selectively reshapes long‐range contact patterns for BBBPPs in a way that is compatible with the formation of transport‐competent conformations. Importantly, the perturbation‐based faithfulness analysis further shows that residues assigned high attribution are more influential to the final prediction than low‐attribution or randomly selected residues, thereby strengthening the link between visualization and model behavior. Although these results remain computational rather than experimental and should not be interpreted as direct proof of biochemical mechanism, they provide stronger evidence that the representations learned by INB^3^P are not only predictive, but also meaningfully aligned with current understanding of BBB‐penetrating peptide design.

### 2.6 Mechanistic Consistency as in Silico Validation

A critical challenge in applying deep learning to biological sequences is distinguishing between genuine mechanistic learning and the overfitting of dataset‐specific noise. Although this study relies on computational validation, multiple interpretability analyses revealed that the model learned representations that are broadly consistent with established biophysical expectations for BBB‐penetrating peptides. These findings should therefore be interpreted as mechanistically supportive in silico evidence, rather than as a substitute for direct experimental validation.

Without explicit supervision on biophysical rules, INB^3^P spontaneously “rediscovered” key design principles of BBB‐crossing. Specifically, the attention mechanism consistently highlighted motifs with balanced cationicity and polarity while suppressing extensive hydrophobic patches, which is consistent with the proposed requirement for an intermediate physicochemical regime that supports membrane interaction without excessive nonspecific disruption. Furthermore, the analysis of contact maps revealed that the model's fine‐tuning stage selectively sharpened long‐range structural contacts for positive samples (Figure 6W,X). This observation suggests that the model is learning structurally informative patterns associated with transport‐competent conformational organization, rather than relying solely on superficial sequence‐level correlations.

Taken together, the consistency between the learned representations and established biochemical principles supports the biological plausibility of the model and strengthens confidence in its prioritization capability in an in silico setting. At the same time, such evidence should not be interpreted as proof of experimental transport activity, stability, or therapeutic efficacy, all of which still require dedicated in vitro and, where appropriate, in vivo validation.

### 2.7 Web Server Implementation and Accessible Augmentation Tools

**FIGURE 8:**
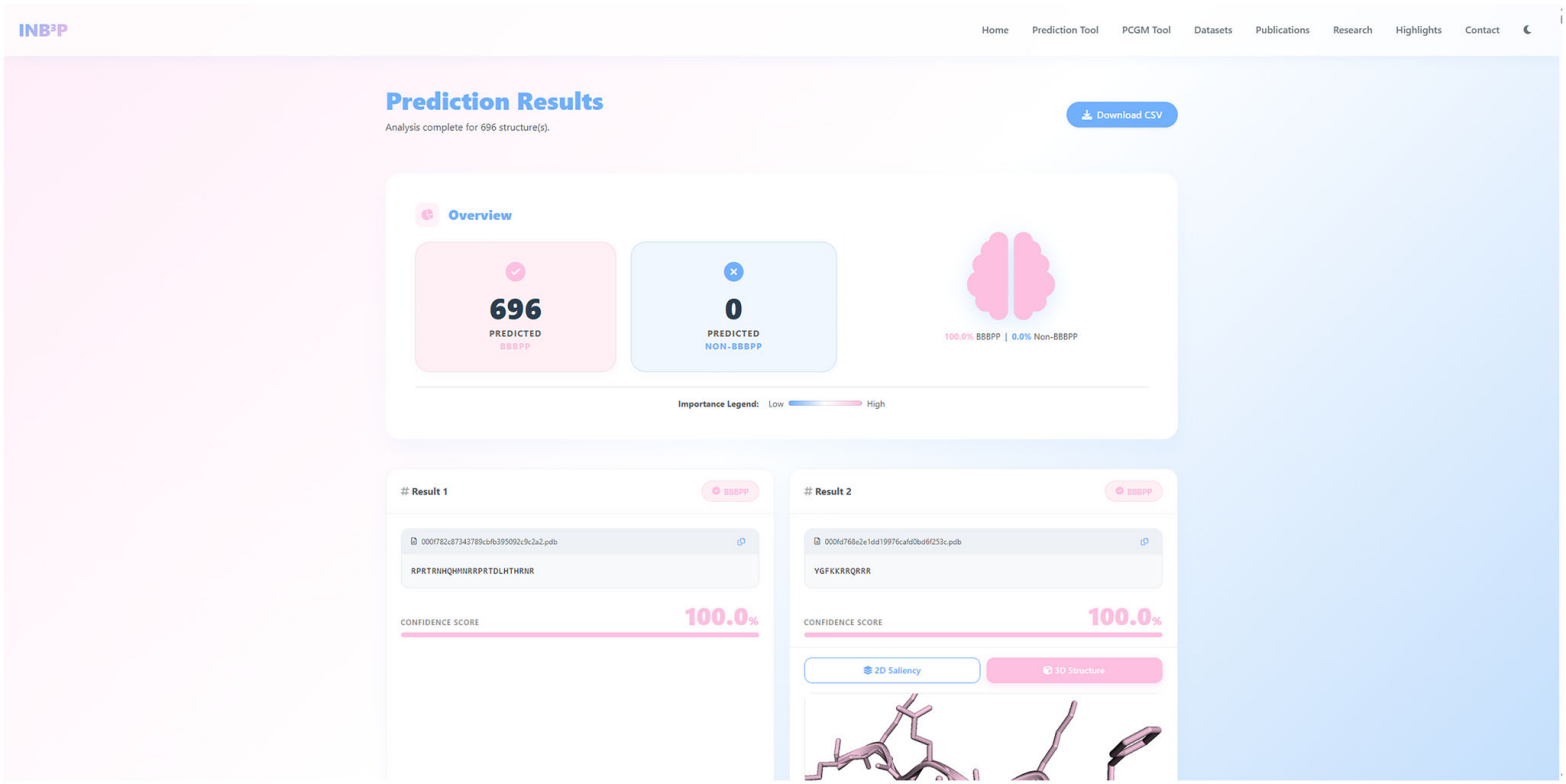
The interactive prediction and interpretability dashboard of the INB^3^P web server. The server supports high‐throughput batch processing, as shown by the overview panel summarizing predictions for 696 input sequences. (Bottom Panels) Beyond binary classification, the interface provides granular mechanistic insights for each peptide. The “Confidence Score” indicates the model's certainty. Crucially, users can toggle between “2D Saliency” and “3D Structure” tabs to inspect the model's decision process. As shown in Result 2, the 3D Structure view visualizes the peptide's conformation, enabling researchers to examine the spatial arrangement of key residues and potential contact motifs described in the study.

**FIGURE 9:**
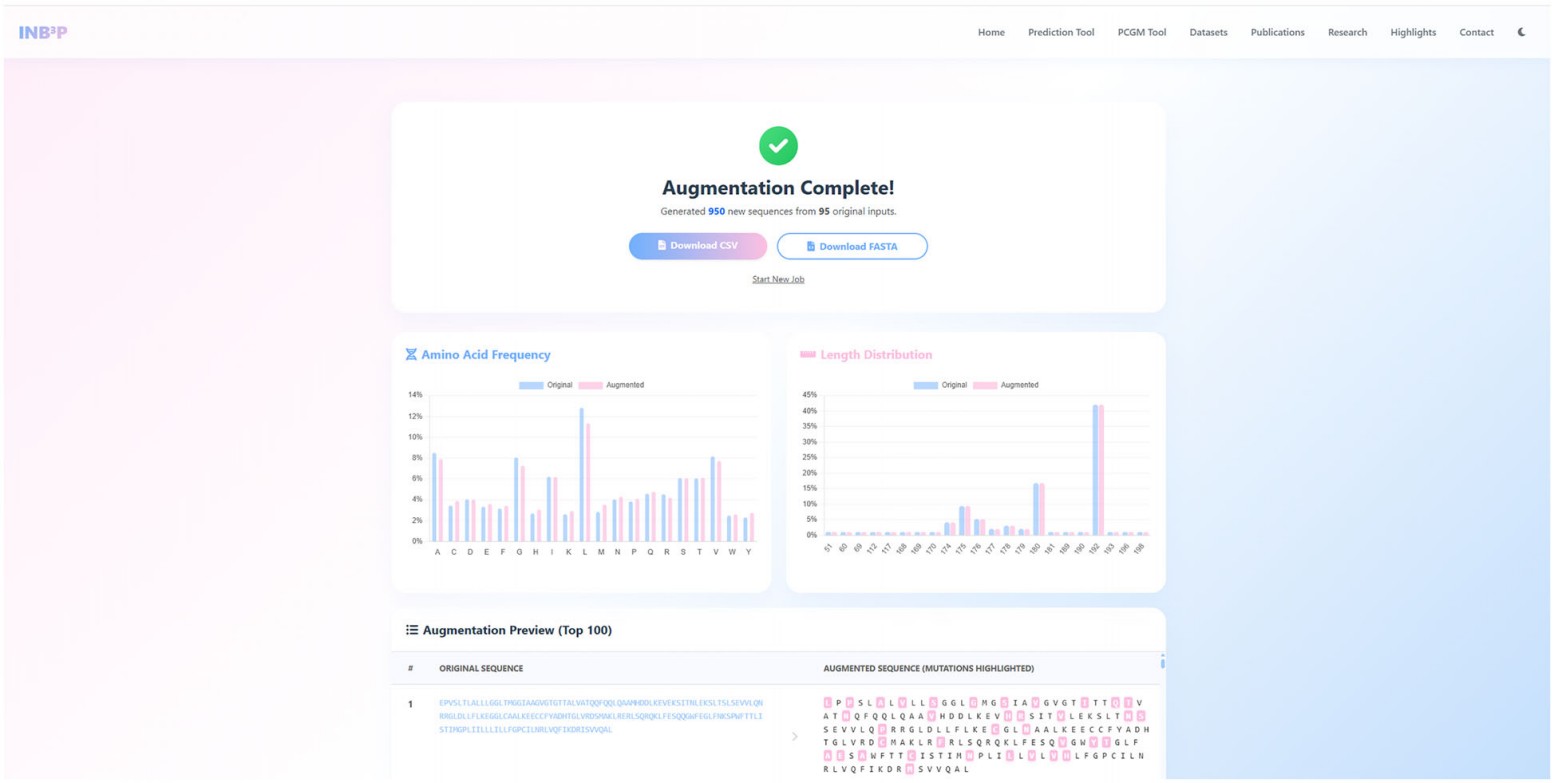
Interface of the standalone PCGM Augmentation Module on the INB^3^P web server. This general‐purpose module allows users to upload custom datasets and perform physicochemical‐guided augmentation. The results dashboard demonstrates the algorithm's fidelity: (Middle Panel) Comparative histograms verify that the augmented sequences (pink) rigorously preserve the global amino acid frequencies and length distributions of the original input (blue), avoiding the statistical distortion common in random augmentation. (Bottom Panel) An interactive preview highlights specific residue substitutions (pink boxes) generated by the algorithm, offering full transparency into the mutation process. Users can download the enriched datasets in CSV or FASTA formats for downstream model training.

To facilitate the practical application of INB^3^P, we developed a comprehensive web server comprising three functional modules. Importantly, beyond returning a binary prediction, the server explicitly reports the raw prediction probability (confidence score) for each queried peptide, thereby enabling user‐side decision control under different screening priorities. First, the Prediction Module accepts peptide sequences and returns both predicted labels and probability scores, alongside interactive visualization of attention weights, allowing users to intuitively identify key residues driving BBB penetration. By exposing continuous prediction scores rather than only dichotomized outputs, the server provides a more transparent basis for practical interpretation, especially in scenarios where users may wish to adopt stricter or more permissive decision thresholds. Second, the structure visualization module displays the predicted contact maps, highlighting the long‐range interactions stabilized by our fine‐tuning process (Figure 8). Third, and most crucially, the PCGM augmentation module is offered as a general‐purpose tool. Recognizing that data scarcity is a universal challenge in peptide therapeutics, we allow users to upload their own datasets and apply the PCGM algorithm with customizable parameters (Figure 9). This empowers the broader community to leverage physically constrained augmentation for diverse functional peptide classes beyond BBBPPs.

## 3 Conclusion

In this work, we introduced INB^3^P, a cross‐modal deep learning framework tailored to the discovery of BBBPPs under extreme class imbalance. By jointly leveraging sequence semantics, predicted three‐dimensional structure, and residue‐level physicochemical context, INB^3^P addresses three key obstacles that have limited previous predictors: the scarcity and heterogeneity of experimentally validated BBBPPs, the dominance of the non‐BBBPP class during optimization, and the lack of mechanistic interpretability in model decisions.

Methodologically, our study makes three main contributions. First, we developed PCGM and a stratified mini‐batch sampler to tackle severe positive‐class scarcity without distorting low‐order sequence statistics. PCGM enlarges the BBBPP training corpus through BLOSUM‐ and property‐aware substitutions that preserve amino‐acid composition and length distributions, while the stratified sampler enforces approximately balanced mini‐batches even when the global ratio of positives to negatives is about 1:20.8. Second, we designed a cross‐modal architecture that fuses ESM‐2–derived sequence representations with ESMFold‐based contact graphs enriched by residue‐level physicochemical features via bidirectional co‐attention and graph transformers. Third, we employed a two‐stage training curriculum that first aligns sequence and structure through multimodal contrastive learning (InfoNCE and supervised contrastive losses) and then fine‐tunes a classifier using an imbalance‐robust objective that combines focal loss with a stable, differentiable MCC loss.

Empirically, INB^3^P achieves substantial and consistent gains over existing BBBPP predictors on both cross‐validation and an independent test set. The model delivers high AUROC and average precision while simultaneously improving sensitivity, specificity, and MCC, particularly enhancing positive‐class detection at stringent false‐positive control compared with prior methods. Ablation experiments demonstrate that each component of the framework contributes to these gains: PCGM and stratified sampling jointly mitigate overfitting and majority‐class dominance; the sequence–structure fusion module outperforms unimodal variants; and the composite focal+MCC loss outperforms either term alone across multiple evaluation metrics. Together, these results suggest that predictive signals associated with BBBPP identification in the current benchmark are genuinely distributed across sequence, structure, and physicochemical patterns, and that INB^3^P can integrate these signals into effective decision boundaries under this dataset setting.

Beyond predictive performance, INB^3^P offers mechanistic interpretability that connects data‐driven representations to biochemical design principles. Property‐level enrichment analyses suggest that the model assigns higher scores to BBBPP candidates with moderate cationicity, balanced polarity, and organized patterns of cationic and aromatic residues, while disfavoring long unbroken hydrophobic stretches or strongly anionic motifs—features broadly consistent with proposed constraints for traversing the blood–brain barrier without nonspecific membrane disruption. Residue‐ and contact‐level explanations further show that, despite the absence of any explicit structural loss, fine‐tuning refines the ESM‐2 structural prior in a class‐dependent way: long‐range contact patterns for BBBPPs become sharper and more coherent across training stages, whereas non‐BBBPPs retain more diffuse connectivity. Taken together, these mechanistic consistencies provide supportive in silico evidence that the model is learning biologically meaningful patterns beyond purely superficial label correlations. However, these findings should be interpreted within the scope of the current benchmark and do not by themselves constitute definitive proof of experimental transport competence.

At the same time, the present study has several limitations that define important directions for future work. First, the evaluation is conducted on a single curated BBB peptide assay dataset with binary labels, which collapses potentially distinct transport mechanisms (e.g., receptor‐mediated vs. adsorptive transcytosis) into one “BBBPP” category and therefore limits mechanistic granularity. In addition, the benchmark relies on a literature‐standard negative control set constructed from UniProt via annotation/keyword‐based exclusion rules rather than random proteome sampling. Although this design improves label confidence and preserves direct comparability with prior BBBPP predictors, it may introduce selection bias and thus constrain how directly benchmark performance reflects deployment‐time generalization. Second, both the benchmark sequences and the current sequence representation backbone are restricted to the 20 canonical amino acids. This is a practical limitation for medicinal peptide design, where non‐natural residues are frequently introduced to enhance BBB permeability, stability, and pharmacokinetic behavior. Because most currently available protein language models (including the ESM‐based encoders used here) are pretrained primarily on canonical amino‐acid sequences, non‐natural residues are typically mapped to an unknown token (e.g., “X”), which may reduce chemical fidelity and predictive reliability. Accordingly, this limitation is not specific to the ESM‐based encoders used here, but instead reflects a broader constraint of current mainstream protein language models. Consistently, the current PCGM augmentation space is also defined over canonical residues and therefore does not explicitly model substitutions involving non‐natural amino acids. Third, although we incorporate state‐of‐the‐art structure prediction, the structural branch remains dependent on in silico models rather than experimentally resolved structures, and the current validation is computational rather than prospective. Accordingly, the present framework should be interpreted as an in silico prioritization tool for candidate screening, rather than as a substitute for direct in vitro or in vivo validation. Fourth, although practical deployment would also benefit from considering synthesis feasibility and synthesis cost within the optimization loop, such information is not available in the current benchmark in a sufficiently systematic and matched form, and therefore could not be incorporated rigorously in the present study. Future studies should therefore expand INB^3^P to larger and more diverse datasets with finer mechanistic annotations, evaluate performance on broader and less constrained negative sets (including experimentally curated hard negatives where available), and test predictions in physiologically relevant in vitro and in vivo BBB models. Additional directions include extending INB^3^P/PCGM toward non‐canonical‐residue‐aware representation learning and augmentation (e.g., expanded tokenization schemes and physicochemical descriptors for modified residues), as well as benchmarking the framework with newer protein foundation models (e.g., ESM‐C and ESM3) as deployment constraints and task‐specific fine‐tuning ecosystems become more mature and reproducible. Finally, because INB^3^P provides a differentiable and interpretable scoring function over sequences, it offers a natural basis for integration with generative models to propose de novo BBBPP candidates, enabling closed‐loop design, screening, and experimental validation. By exposing both the INB^3^P scoring function and the PCGM augmentation module through a web‐accessible interface, we further provide a foundation for human‐in‐the‐loop peptide optimization, in which experimentalists can rapidly screen candidates, interpret model decisions, and iteratively refine sequences using biophysical feedback.

In summary, INB^3^P provides a state‐of‐the‐art framework for BBBPP prediction and establishes a broader methodological direction for small‐sample biological sequence learning. In particular, the PCGM augmentation strategy and the imbalance‐aware Stable‐MCC optimization scheme may be transferable to other peptide prediction settings characterized by limited labeled data and skewed class distributions. More broadly, we hope that the principles developed here will prove useful for related functional peptide tasks, such as antimicrobial or tumor‐homing peptide prediction, and will contribute to a closer integration of data‐driven modeling with mechanistically informed peptide discovery.

## 4 Materials and Methods

### 4.1 The Blood–Brain Barrier Penetrating Peptides Dataset and Data Argumentation

In this study, we used the benchmark dataset curated by Tang et al. [2]. Building on the earlier BBPpredict release [17], Tang et al. expanded the positive set by integrating BBB‐penetrating peptides from public resources beyond the original compilation, whose upstream positives were derived from the literature‐curated B3Pdb database. The negative control set was constructed using the BBPpredict UniProt filtering protocol [37], excluding BBB/brain‐related and membrane/transport‐related entries, followed by quality‐control filtering (reviewed entries, 5–50 aa, no ambiguous residues) and redundancy reduction using CD‐HIT at 40% identity [38]. For strict comparability with prior BBBPP predictors, we used the released DeepB3P train/test partitions as‐is, without re‐splitting or relabeling. The final corpus comprised 329 BBBPPs and 6851 non‐BBBPPs for training and 99 BBBPPs plus 99 non‐BBBPPs for testing (Table 6). Because these labels were aggregated from heterogeneous source studies and were not explicitly harmonized for inter‐platform variability at the benchmark level, our results should be interpreted primarily as comparative benchmark performance. Additional details on label provenance and benchmark construction are provided in Note S2.

**TABLE 6:** Details of samples in the blood–brain barrier penetrating peptides dataset.

Dataset	Positive samples	Negative samples	Ratio
Training set	329	6851	1:20.8
Independent test Set	99	99	1:1

Given the severe class imbalance in our training data and that Tang et al. used a feedback GAN to achieve state‐of‐the‐art performance, we sought a more lightweight and controllable alternative to sequence generation. We introduce PCGM, which perturbs minority‐class peptides via constrained substitutions that preserve biochemical plausibility while increasing sample diversity and decision margins.

Let a peptide be *S*  =  (*a*_1_,…, *a_L_*) with residues $a_{i}\in A$, where A={A,C,…,Y} denotes the 20 canonical amino acids. PCGM proposed a small number of amino‐acid substitutions to produce an augmented peptide $\tilde{S}$. Unlike uniform or purely BLOSUM‐driven schemes, PCGM fused evolutionary plausibility with physicochemical similarity. Below is the mathematical formulation and substitution distributions. We also provide a step‐by‐step pseudocode appears in the (Algorithm S1).

### 4.2 Property Embedding and Normalization

In PCGM, each residue x∈A Is Mapped to a *d*‐dimensional property vector.

(3) $$p\;\left(x\right)={\left[p^{\left(1\right)}\left(x\right),\text{…},p^{\left(d\right)}\left(x\right)\right]}^{⊤}\;,d=9$$

which covering nine physicochemically meaningful properties (the detailed definitions and references are shown in Table S14). We then z‐scored each dimension across A in order to place heterogeneous properties on a common scale, preventing any single high‐magnitude feature from dominating distance or attention computations, made the radial basis function (RBF) kernel bandwidth τ interpretable in standard‐deviation units, stabilized optimization, and cross‐modal alignment also ensured reproducible, leakage‐free statistics independent of the training data.

(4) $$\hat{p}{\left(x\right)}_{k}=\frac{p^{\left(\kappa \right)}\left(x\right)-\mu_{\kappa }}{\sigma \kappa },\mu_{\kappa }=\frac{1}{\left|A\right|}\sum_{x\in A}p^{\left(\kappa \right)}\left(x\right),\sigma_{\kappa }^{2}=\frac{1}{\left|A\right|}\sum_{x\in A}{\left(p\left(\kappa \right)-\mu_{\kappa }\right)}^{2}$$

#### 4.2.1 Affinity Kernels

Let A denote the 20 canonical amino acids and let $\hat{p}\left(x\right)\in R^{9}$ be the z‐scored physicochemical vector for residue *x* (computed once over A). We used two complementary affinities to quantify how plausible a substitution *x* →  *y* is from a physicochemical and an evolutionary standpoint.

#### 4.2.2 Physicochemical

In the normalized property space, residues that are similar in hydropathy, charge, size, polarity, etc., should be closer. We therefore used a Gaussian (RBF) affinity.

(5) $$K_{\mathrm{prop}\;}\left(x,y\right)=\;\exp\left(-\frac{∥\hat{p}\left(x\right)-\hat{p}\left(y\right){∥}_{2}^{2}}{2\tau^{2}}\right),\;\tau >0$$

where the bandwidth τ controls tolerance to property drift (small τ enforces stricter similarity while large τ permits broader substitutions). Because each property is z‐scored across A, τ is interpreted.

#### 4.2.3 BLOSUM‐Derived Kernel

Evolutionary plausibility was captured by BLOSUM [39, 40] log‐odds scores *B*(*x*, *y*). We converted these scores into a row‐stochastic affinity over candidate substitutions from *x* by a temperature‐scaled SoftMax:

(6) $$K_{\mathrm{blo}\;}\;\left(x,y\right)=\frac{\exp\left(\beta B\left(x,y\right)\right)}{\sum_{z\in A}\exp\left(\beta B\left(x,z\right)\right)}\;,\;\beta >0\;$$

so that $\sum_{y}K_{\mathrm{blo}}\left(x,y\right)=1$ for all *x*. The scale β (inverse temperature) sharpens or flattens the evolutionary prior; higher β emphasizes high‐BLOSUM substitutions, while lower β spreads mass more uniformly. *K*_prop_ is symmetric by construction; *K*_blo_ can be asymmetric, reflecting directionality in empirical substitution tendencies. Both tables are precomputed once over A×A and cached.

#### 4.2.4 Fusion and Sampling Distribution

To combine complementary evidence, we first normalized both affinity matrices to the [0,  1] range and then fused them via a linear combination with weights *w_blo_* and *w*_prop _:

(7) $$\tilde{K}\left(x,y\right)=w_{blo}\;\;\times \;{\hat{K}}_{blo}\left(x,y\right)+w_{prop}\;\times \;{\hat{K}}_{prop}\left(x,y\right)$$

where $\tilde{K}$ denotes the min–max normalized affinity matrices.

A temperature‐controlled SoftMax then yielded a substitution distribution:

(8) $$P_{\mathrm{sub}\;}\;\left(y|x\right)=\frac{\tilde{K}{\left(x,y\right)}^{\frac{1}{T}}}{\sum_{z\in A}\tilde{K}{\left(x,z\right)}^{\frac{1}{T}}}\;,\;T\;>0\;$$

where smaller *T* produces peakier proposals. Optionally, identity substitutions can be forbidden by setting $\tilde{K}\left(x,x\right)=0$ before normalization.

#### 4.2.5 Mutation Policy

For each peptide *S*, PCGM first decided whether to apply augmentation at the sample level with probability *p*_aug_; if not triggered, the peptide remained unchanged. When augmentation was triggered, positions were considered independently for mutation via Bernoulli draws with probability *p*_mut_. To limit perturbation strength, we enforced a hard budget *M*_max_  =  |ρ_max_ *L*| mutated sites per peptide; if the number of selected sites exceeded this cap, we randomly retained exactly *M*_max_ of them and reverted the rest. Substitutions at the retained indices were sampled from the fused substitution distribution *P*_sub_(· ∣*a_i_*), constructed from BLOSUM‐based and property‐based affinities with optional identity‐forbidding and temperature control; unselected positions were left unchanged. The result was an augmented sequence $\tilde{S}$ that preserves length and applies a small, physicochemically plausible set of edits.

#### 4.2.6 Two‐Stage Curriculum

We adopted a mild two‐stage curriculum [41]. In Stage‐1 (representation learning), augmentation was active with a tuple of controls $\left(p_{\mathrm{aug}}^{\left(1\right)},p_{\mathrm{mut}}^{\left(1\right)},\rho_{\max}^{\left(1\right)}\right)$ and a short warm‐up over the first *E*_warm_ epochs, during which the sample‐level augmentation probability *p*_aug _ was gradually ramped from zero to its target value $p_{\mathrm{aug}}^{\left(1\right)}$, while the mutation intensity parameters ($p_{\mathrm{mut}}^{\left(1\right)},\rho_{\max}^{\left(1\right)})$ were applied at their full target values whenever augmentation was triggered.

In Stage‐2 (supervised fine‐tuning), augmentation remained enabled with its own tuple $(p_{\mathrm{aug}\;}^{\left(2\right)},p_{\mathrm{mut}\;}^{\left(2\right)},\rho_{max}^{\left(2\right)}),$ chosen to be more label‐faithful than Stage‐1 ($p_{\mathrm{mut}}^{\left(2\right)}\leq p_{\mathrm{mut}}^{\left(1\right)}$ and $\rho_{\max}$ not exceeding the Stage‐1 budget), while keeping coverage high ($p_{\mathrm{aug}}^{\left(2\right)}$ not smaller than $p_{\mathrm{aug}}^{\left(1\right)}$). Temperature and fusion weights (*T*, α) as well as kernel bandwidth and scales (τ, β) were held fixed or tuned per stage as (*T* ^(*s*)^,α^(*s*)^,τ^(*s*)^,β^(*s*)^) with *s* ∈ {1, 2}, depending on validation stability. Identity‐forbidding and logging policies remain consistent across stages.

This staged design was adopted because the contrastive alignment objectives in Stage‐1 and the classification‐oriented objectives in Stage‐2 play different optimization roles: the first stage stabilized multimodal representation learning under augmentation and severe class imbalance, whereas the second stage refined the decision boundary for the final prediction task. In preliminary experiments, directly optimizing all objectives from the beginning led to less stable training and weaker convergence of the cross‐modal representations, so we used a mild two‐stage curriculum to decouple representation shaping from task‐specific classification refinement. A more detailed rationale for this design is provided in Note S3.

#### 4.2.7 Monitoring and Reproducibility

For each Mutation $a_{i}\to {\tilde{a}}_{i}$, We record the property delta

(9) $$\Delta p_{i}=\hat{p}\;\left({\tilde{a}}_{i}\right)-\hat{p}\left(a_{i}\right)$$

and track ∥Δ**p**_*i*_∥_2_, per‐dimension shifts, and correlation with BLOSUM to verify physicochemical plausibility. We seeded the per‐sample random number generator (RNG) with a hash of (RUN_ID,epoch,global_index) for determinism. When the environment variable AUG_LOG was set, PCGM wrote CSV logs of $\left(i,a_{i},{\tilde{a}}_{i},\Delta p_{i}\right)$ and aggregated summaries (means, histograms, top‐k substitutions).

### 4.3 Stratified Mini‐Batch Sampling Under Class Imbalance

Even after augmentation, the training corpus remained globally imbalanced. To prevent stochastic gradient updates from being dominated by the majority class, we implemented a custom stratified mini‐batch sampler (STRATIFIED‐BATCH‐SAMPLER). At the start of training, all indices were partitioned into positive (BBBPP) and negative (non‐BBBPP) pools. For each training step, the sampler drew a mini‐batch of fixed size *B* by selecting ⌊*B*/2⌋ positives and *B* − ⌊*B*/2⌋ negatives whenever possible. When the number of available positives was insufficient to fill a batch (which is common given the 1:20.8 ratio), the sampler resorts to sampling with replacement from the positive pool, whereas negatives were drawn without replacement until their pool was exhausted. The indices within each batch were then randomly permuted so that the classifier did not see a fixed class ordering.

This stratified mini‐batch construction was used consistently in both Stage 1 and Stage 2 and treated augmented BBBPPs as positives. By enforcing an approximately balanced class composition at the batch level, STRATIFIED‐BATCH‐SAMPLER ensured that the classifier received informative gradients from both classes at every update step, stabilized optimization of our imbalance‐aware loss (a weighted combination of focal loss and a differentiable MCC surrogate), and reduced sensitivity to fluctuations in the global class ratio or to the exact choice of augmentation hyperparameters. Random seeds were fixed for the sampler to guarantee reproducibility of batch composition across runs. Full pseudocode for STRATIFIED‐BATCH‐SAMPLER is provided in Algorithm S3.

### 4.4 Model Architecture

Prior studies indicated that incorporating physicochemical descriptors could complement ESM‐2–derived sequence representations at mean time BBPPs often exhibited characteristic structural. Motivated by these observations, we proposed a multi‐modal framework that fused evolutionary and contextual representations extracted by ESM‐2 with structural features inferred by ESMFold, while enriching residue nodes with a compact set of physicochemical attributes. Because ESMFold was integrated within the pipeline, the model required only the raw amino‐acid sequence as input. The overall multimodal architecture (Figure 1).

#### 4.4.1 Sequence Encoder (ESM2 [31, 42])

To capture long‐range residue–context dependencies relevant to uptake and translocation, a peptide *S*  =  (*a*_1_,…, *a_L_*)is encoded with ESM‐2 to obtain contextual token embeddings

(10) $$H^{seq}=\left[h_{1},\text{…},h_{L}\right]\;\in R^{L\times d_{s}}$$

A lightweight projection aligned channel width with the structural stream:

(11) $$S=H^{\mathrm{seq}}\;W_{s}+b_{s},\;W_{s}\in R^{d_{s}\times d}$$

This preserved biological priors distilled by language modeling while placing both modalities in a shared latent space for fusion.

#### 4.4.2 Residue Graph Construction and Structural Encoder

Because BBB traversal depends on 3D presentation, like exposure of charged and hydrophobic motifs, we represented each peptide as a residue contact graph *G*  =  (*V*, *E*) with *V*  =  {1, …, *L*} and edges between spatially proximal residues, C_α_ distance ≤ *r*. Residue *i* is described by

(12) $$x_{i}=\left[\mathrm{onehot}\left(a_{i}\right)∥\hat{p}\left(a_{i}\right)\right]\in R^{29}$$

where $\hat{p}\left(\cdot \right)\in R^{9}$ is a z‐scored physicochemical vector. Distance‐aware edge features ϕ_*ij*_ are encoded with an RBF bank to modulate message passing. Edge‐aware multi‐head attention stack aggregated neighborhood evidence while respecting geometry:

(13) $$e_{ij}=\frac{\left(x_{i}W_{Q}\right){\left(x_{j}W_{K}\right)}^{⊤}}{\sqrt{d_{h}}}+\phi_{ij}\cdot w_{\phi },\;$$

(14) $$\alpha_{ij}=\mathrm{softma}x_{j\in N\left(i\right)}\left(e_{ij}\right)\;,$$

(15) $$g_{i}=\sum_{j}a_{ij}\left(x_{j}w_{v}\right)$$

yielding residue‐level embeddings

(16) $$H^{\mathrm{pdb}}=\left[g_{1},\text{…},g_{L}\right]\;\in R^{L\times d}$$

Maintaining residue resolution allowed later fusion to align specific tokens to specific structural locales, which was essential for interpretability.

#### 4.4.3 Bi‐Directional Cross‐Modal Co‐Attention (Fusion Block)

Sequence and structure encode complementary inductive biases: composition versus conformation. To couple them without collapsing one into the other, we used bi‐directional co‐attention that aligns tokens to residues and residues back to tokens within each block. With learned projections, the Seq → PDB update was

(17) $$\mathrm{Att}n_{s\to p}\left(S,P\right)=\mathrm{softmax}\left(\frac{\left({\mathrm{SW}}_{Q}^{s}\right){\left({\mathrm{PW}}_{K}^{p}\right)}^{⊤}}{\sqrt{d_{h}}}+\log M\right)\;\left({\mathrm{PW}}_{V}^{p}\right)$$

and the PDB → Seq path swaps (**S**, **P**). The mask **M** zeroes padded or invalid alignments. Residual connections, layer normalization, and position‐wise feed‐forward layers stabilized optimization and permitted stacking *L_f_* blocks. Practically, these blocks surface cross‐modal saliency maps that pinpoint structural neighborhoods explaining sequence features implicated by the language model, producing fused yet decomposable representations.

(18) $${\tilde{H}}^{\mathrm{seq}},{\tilde{H}}^{\mathrm{pdb}}\in R^{L\times d}$$

#### 4.4.4 Masked Pooling and Modality Embeddings

Downstream decisions required sequence‐level and structure‐level summaries while retaining a path back to residues. We therefore applied length‐aware masked means,

(19) $$s=\frac{1}{\sum_{i}m_{i}^{\mathrm{seq}}}\;\sum_{i=1}^{L}m_{i}^{\mathrm{seq}}{\tilde{h}}_{i}^{\mathrm{seq}},p=\frac{1}{\sum_{i}m_{i}^{\mathrm{pdb}}}\;\sum_{i=1}^{L}m_{i}^{\mathrm{pdb}}{\tilde{h}}_{i}^{\mathrm{pdb}}$$

to obtain modality descriptors that were insensitive to padding yet traceable to residue contributions via the masks. Small projection heads then placed these descriptors on a unit sphere,

(20) $$z_{s}=\frac{\psi_{s}\left(s\right)}{∥\psi_{s}\left(s\right){∥}_{2}}\;,\;z_{p}=\frac{\psi_{p}\left(p\right)}{∥\psi_{p}\left(p\right){∥}_{2}}\;$$

facilitating well‐behaved similarity measures across modalities and enabling analyses such as cross‐modal retrieval or calibration of sequence–structure consistency.

#### 4.4.5 Fusion Readout (Classification Interface)

To translate joint evidence into a scalar decision while remaining modular, we fused the pooled descriptors and applied a compact readout:

(21) $$f\;=\phi \left(\left[s∥p\right]\right)\in R^{d},o=w^{⊤}\;f+b,\;\hat{y}=\sigma \left(o\right)$$

This interface cleanly separated representation learning from the final decision layer, allowing threshold selection and calibration to proceed independently in evaluation, and enabling ablations that isolate the contribution of fusion versus unimodal encoders.

#### 4.4.6 Explainability Interface (Token ↔ Residue Attribution)

Interpretability is achieved not by post‐hoc heuristics alone but by design choices that preserve alignment signals. Each fusion block exposes attention tensors **A**_*s* → *p*_, **A**_*p* → *s*_ that can be rolled out across layers and combined with gradient‐based scores to attribute predictions back to residues and tokens:

(22) $$s^{\mathrm{attr}}=\sum_{\ell =1}^{L_{f}}\mathrm{rollout}\left(A_{s\to p}^{\ell }\right)⊙|\frac{\partial \hat{y}}{\partial {\tilde{H}}^{\mathrm{seq},\left(\ell \right)}}|,$$

(23) $$p^{\mathrm{attr}}=\sum_{\ell =1}^{L_{f}}\mathrm{rollout}\left(A_{s\to p}^{\left(\ell \right)}\right)⊙|\frac{\partial \hat{y}}{\partial {\tilde{H}}^{\mathrm{pdb},\left(\ell \right)}}|$$

Because these maps respect the explicit token–residue correspondence induced by co‐attention, they can be compared to contact patterns or colored by physicochemical annotations, thereby revealing why specific sequence segments and structural neighborhoods support BBB‐crossing behavior and enabling residue‐level design suggestions.

### 4.5 Loss Functions

To obtain robust cross‐modal alignment and reliable classification under class imbalance, this model was trained with a two‐stage composite objective. Let sample *i* have a label. *y_i_* ∈ {0, 1}, logit *s_i_*, probability *p_i_* =  σ(*s_i_*). We applied label smoothing to the hard target.

(24) $${\tilde{y}}_{i}=\left(1-\varepsilon \right)\;y_{i}+0.5\varepsilon$$

used class weights (*w_p_*,*w_n_*) for positives and negatives, also denoted the focusing parameter by γ  >  0. Temperatures for InfoNCE and supervised contrastive learning (SCL) are *T*  >  0 and τ  >  0. A small constant δ  >  0 ensured numerical stability in MCC.

#### 4.5.1 Stage 1: Representation Learning (Alignment First, Supervision Later)

Stage‐1 trains the sequence encoder, the PDB‐graph encoder, and fusion blocks to learn informative, aligned representations, while gradually introducing supervision. With linear curriculum weights *w*_con_ =  1 − progress and *w*_sup_ =  progress,

(25) $$\begin{array}{ccc} L^{\left(1\right)} & = & w_{\mathrm{con}\;}\;\left[\lambda_{\mathrm{con}\;}L_{\mathrm{InfoNCE}\;}+\lambda_{\mathrm{scl},\;s}L_{\mathrm{SCL}\;}^{\left(\mathrm{seq}\;\right)}+\lambda_{\mathrm{scl},\;p}L_{\mathrm{SCL}\;}^{\left(\mathrm{pdb}\;\right)}\right]\\ & & +w_{\sup\;}\lambda_{\mathrm{cls}1\;}\left(\omega_{f}L_{\mathrm{Focal}\;}+\omega_{m}L_{\mathrm{MCC}\;}\right) \end{array}$$

#### 4.5.2 Cross‐Modal InfoNCE [43]

For L2‐normalized embeddings $\left(z_{i}^{\mathrm{seq}},z_{i}^{\mathrm{pdb}}\right)$, the within‐pair was the positive; negatives came from the current batch and a momentum memory bank. Only the top‐*k* most challenging negatives entered the SoftMax. Using dot products 〈 ·, ·〉 (cosine‐equivalent),

(26) $$\begin{array}{ccc} L_{\mathrm{InfoNCE}\;} & = & \frac{1}{2B}\;\sum_{i=1}^{B}[\mathrm{CE}\left(\left[\langle z_{i}^{\mathrm{seq}\;},z_{i}^{\mathrm{pdb}\;}\rangle /T,\;\mathrm{top}-\;k\langle z_{i}^{\mathrm{seq}\;},K_{p}\rangle /T\right],0\right)\\ & & +\mathrm{CE}\left(\left[\langle z_{i}^{\mathrm{pdb}\;},z_{i}^{\mathrm{seq}\;}\rangle /T,\;\mathrm{top}-\;k\langle z_{i}^{\mathrm{pdb}\;},K_{s}\rangle /T\right],0\right)] \end{array}$$

#### 4.5.3 SCL [44]

To enforce class separability within each modality, we applied SCL on the sequence and structure branches independently. For features *f_i_* and labels *y_i_*, with positive set *P*(*i*)  =  {*j* ≠ *i*:   *y_j_* = *y_i_* },

(27) $$\begin{array}{c} L_{\mathrm{SCL}}\;\left(f,y\right)=-\frac{1}{\left|A\right|}\sum_{i\in A}\frac{1}{\left|P\left(i\right)\right|}\sum_{j\in P\left(i\right)}\log\frac{\exp\left(\frac{\langle f_{i},f_{j}\rangle }{\tau }\right)}{\sum_{k\neq i}\exp\left(\frac{\langle f_{i},f_{k}\rangle }{\tau }\right)} \end{array}$$

This implementation removed self‐pairs, applied per‐row max subtraction for stability, and guarded anchors without positives.

#### 4.5.4 Stage 2: Supervised Fine‐Tuning (Classification Only)

Stage‐2 dropped contrastive terms and focused on supervised classification with a composite of focal and differentiable MCC:

(28) $$L^{\left(2\right)}=\omega_{f}\;L_{\mathrm{Focal}\;}+\omega_{m}L_{\mathrm{MCC}}$$

This pairing was crucial under severe imbalance: focal emphasized hard instances locally, while MCC directly balanced global error trade‐offs (*TP*, *TN*, *FP*, *FN*) and aligned training with the final, threshold‐agnostic evaluation target.

#### 4.5.5 Focal Loss [45]

We computed per‐example BCE on logits against the smoothed target ${\tilde{y}}_{i}$, then applied the focal modulator (1 − *p*_*t*,*i*_)^γ^ and class weights:

(29) $$L_{\mathrm{Focal}\;}=\frac{1}{B}\;\sum_{i=1}^{B}\alpha_{i}\underset{\mathrm{on}\;\;\mathrm{smoothed}\;\;\mathrm{target}\;\;{\tilde{u}}_{i}}{\underset{︸}{\mathrm{BCEWithLogits}\left(s_{i},{\tilde{y}}_{i}\right)}}{\left(1-p_{t,i}\right)}^{\gamma },$$

(30) $$p_{t,i}=p_{i}\;y_{i}+\left(1-p_{i}\right)\left(1-y_{i}\right),\;\;\;\;\alpha_{i}=\{\begin{array}{cc} w_{p}, & \;y_{i}=1\\ w_{n}, & \;y_{i}=0 \end{array}$$

While tuning α and γ mitigates imbalance, focal alone optimizes instance‐wise likelihood and can yield unstable precision–recall trade‐offs under extreme skew; hence, it was combined with MCC.

#### 4.5.6 Stable, Differentiable MCC (Soft Counts With Per‐Factor Stabilization)

We clamped probabilities *p_i_* ∈ [δ, 1 − δ] and used smoothed targets to form soft counts with class weights:

(31) $$\begin{array}{ccc} \mathrm{TP} & = & \sum_{i}w_{p}{\tilde{y}}_{i}p_{i},\mathrm{TN}=\sum_{i}w_{n}\left(1-{\tilde{y}}_{i}\right)\left(1-p_{i}\right),\\ & & \mathrm{FP}=\sum_{i}w_{n}\left(1-{\tilde{y}}_{i}\right)p_{i},\mathrm{FN}=\sum_{i}w_{p}{\tilde{y}}_{i}\left(1-p_{i}\right) \end{array}$$

The soft MCC and its loss are

(32) $$MCC=\frac{TP\;\times \;TN-FP\;\times \;FN}{\sqrt{\left(TP+FP+\delta \right)\left(TP+FN+\delta \right)\left(TN+FP+\delta \right)\left(TN+FN+\delta \right)}+\delta }\;$$

(33) $$L_{MCC}=1-\mathrm{MCC}\;$$

where each factor in the denominator is individually stabilized by + δ, and the overall denominator receives an additional + δ. This mirrors the implementation, preserves differentiability (no hard thresholds), and prevented divide‐by‐zero. The pseudocode appears in the (Algorithm S3).

### 4.6 Evaluation Metrics and Threshold Selection

To compare prior baselines and to rigorously assess performance under class imbalance, we reported both threshold‐based statistics and threshold‐free area measures. Let the confusion‐matrix counts be: true positives (*TP*), true negatives (*TN*), false positives (*FP*), and false negatives (*FN*). Unless stated otherwise, metrics were computed on held‐out data. For threshold‐dependent metrics, binary predictions were derived using a fixed decision threshold of 0.5 in the benchmark setting. This cutoff was adopted as a standard and interpretable decision boundary for binary classification and was retained throughout evaluation to ensure consistency and fair comparison with prior BBBPP predictors assessed on the same public dataset.

#### 4.6.1 Threshold‐Based Metrics [46]

(34) $$ACC=\frac{TP+TN}{TP+TN+FP+FN}$$

(35) $$Sn\;\left(\mathrm{Recall}\right)=\frac{TP}{TP+FN}\;$$

(36) $$Sp\;\left(\mathrm{Specificity}\right)=\frac{TN}{TN+FP}\;$$

(37) $$MCC=\frac{\left(TP\times TN\right)-\left(FP\times FN\right)}{\sqrt{\left(TP+FP\right)\left(TP+FN\right)\left(TN+FP\right)\left(TN+FN\right)}}\;$$

#### 4.6.2 Threshold‐Free Curves and Areas

ROC curve: plots true positive rate (*TPR *= Sn) versus false positive rate (*FPR* = FP/(FP  +  TN)).

The ROC‐AUC is the area under this curve; it reflects ranking quality independent of any single threshold.

Precision–recall (*PR*) curve: plots precision versus recall (*Sn*).

The average precision (AP) summarized the PR curve as the area under the precision as a function of recall. In practice, *AP* is computed as

(38) $$\mathrm{AP}=\sum_{n}\left(R_{n}-R_{n-1}\right)P_{n}$$

where (*R_n_*,*P_n_*) traverse the PR curve after applying the standard precision envelope.

Because positive examples were rare, ROC‐AUC and AP provided threshold‐free assessments of ranking performance, while MCC offered a single‐threshold summary that remained sensitive to all error types. Together with ACC, Sn, Sp, and Precision, these metrics provided a comprehensive and comparable view across balanced and imbalanced regimes.

### 4.7 Methodological Landscape Beyond BBBPP Prediction

The discovery of therapeutic and functional peptides (e.g., antimicrobial, anticancer, and cell‐penetrating peptides) was frequently constrained by limited labeled data, sequence redundancy, and the limited interpretability of high‐capacity predictive models [47, 48]. Although the present work focused on BBBPPs, these methodological challenges were not unique to BBBPP prediction. Rather, they were broadly shared across peptide sequence learning tasks, particularly when biologically active motifs were rare, datasets were small or imbalanced, and mechanistic interpretation was required for downstream validation.

### 4.8 Data Scarcity and Augmentation in Peptide Prediction

To mitigate data scarcity in peptide modeling, prior studies had explored a range of augmentation strategies, including random mutation [49], substitution‐based perturbation (e.g., matrix‐guided residue replacement) [50], resampling [51], and generative approaches such as variational autoencoders, generative adversarial networks, and language‐model‐based sequence generation [52]. While these methods could increase apparent sample diversity, unconstrained perturbations might produce biologically implausible variants, and generative approaches might exhibit reduced fidelity when the functional sequence manifold was sparsely sampled [53].

In this context, INB^3^P introduced PCGM as a directed augmentation strategy that constrains sequence perturbation using biophysical priors. PCGM employed a fused substitution kernel that combines evolutionary substitution preferences (BLOSUM62) with a physicochemical similarity matrix defined over nine properties: hydropathy, molecular weight, pI, net charge at pH 7, aromaticity, SASA, molar refractivity, interfacial hydrophobicity, and TPSA. A temperature‐controlled SoftMax was applied to the fused affinity matrix to bias substitutions toward conservative mutations, thereby aiming to preserve biochemical plausibility while expanding sequence diversity in underrepresented classes.

### 4.9 Multimodal Modeling in Comparable Peptide Tasks

Recent peptide prediction frameworks increasingly integrate multiple sources of information, including primary sequence representations, handcrafted physicochemical descriptors, protein language model (PLM) embeddings (e.g., ESM‐family models), and in some cases, structure‐derived priors. In many existing studies, multimodal information was combined through feature concatenation or relatively simple gating schemes [54]. Although effective in some settings, such approaches might not fully capture fine‐grained correspondence between sequence positions and structural context [55].

To address this limitation, our framework adopted a bidirectional cross‐modal co‐attention mechanism to model sequence–structure interactions more explicitly. The *Seq* → *PDB* branch aligned sequence‐level semantic representations with structural neighborhoods, whereas the *PDB* → *Seq* branch contextualizes structural features using sequence information. This mutual conditioning was intended to improve the representation of function‐relevant motifs that depend jointly on sequence composition and structural presentation. In addition, we employed a stratified batch sampling strategy to reduce gradient dominance from majority classes in skewed training distributions, which was particularly important in small‐sample peptide datasets.

### 4.10 Interpretability in Peptide Sequence Prediction

Interpretability in peptide prediction has traditionally relied on post hoc attribution methods (e.g., SHAP‐ or saliency‐based analyses) to identify influential inputs. However, such methods do not always provide mechanistically informative explanations in a biophysical context [56]. More recent efforts have therefore incorporated attention visualization, residue‐level perturbation analyses, and in silico mutagenesis to better link model outputs to sequence motifs and residue preferences.

INB^3^P complements predictive modeling with a multi‐level interpretability analysis pipeline that examines model behavior across multiple resolutions. First, latent‐space visualization (e.g., t‐SNE) was used to assess class separability following multimodal fusion and representation refinement. Second, property‐aware analyses aggregate attribution or attention patterns over physicochemical dimensions to identify biophysically consistent drivers of prediction. Third, structure‐aware analyses examine fine‐tuning‐induced changes in internal contact patterns to evaluate whether the model increasingly emphasizes conformational cues associated with transport‐relevant peptide behavior. Collectively, these analyses improved the biological interpretability and practical transparency of the framework.

### 4.11 Positioning of the Present Study

Relative to prior peptide prediction studies, the contribution of INB^3^P lay in the integration of three methodological components within a unified BBBPP‐oriented framework: (i) a physicochemical‐guided augmentation strategy for small‐sample learning, (ii) explicit multimodal sequence–structure interaction modeling via bidirectional co‐attention, and (iii) a multi‐level interpretability pipeline that links predictive signals to biochemical and structural cues. Although the present study was evaluated on BBBPP prediction, the underlying design principles might be relevant to other peptide prediction tasks that face similar constraints in data availability, class imbalance, and interpretability.

## Fundings

The work is supported by the National Natural Science Foundation of China (No. 62450002, 62572156) and the Science and Technology Development Fund of Macau (no. 0177/2023/RIA3).

## Conflicts of Interest

The authors declare no conflicts of interest.

## Supporting information




**Supporting File**: advs75083‐sup‐0001‐SuppMat.docx.

## Data Availability

The INB^3^P web server, comprising the predictor, interpretability dashboard, and the standalone PCGM augmentation tool, is freely available at http://www.bioai‐lab.com/INBP. The source code and training logs are available to researchers and developers at https://github.com/EuclidLv/INB‐P. The hyperparameters and training commands used in this study are detailed in the Supporting Information. The Zenodo archive containing the materials provided to the reviewers during peer review is now publicly available at:https://zenodo.org/records/17667996
