# Supplementary material for: INB3P: A Multi‐Modal and Interpretable Co‐Attention Framework Integrating Property‐Aware Explanations and Memory‐Bank Contrastive Fusion for Blood–Brain Barrier Penetrating Peptide Discovery
Source: Adv Sci (Weinh). 2026 Apr 3;13(34):e23984. doi: 10.1002/advs.202523984 (PMC13285127; doi:10.1002/advs.202523984)
Supplement: Supplementary file 1 — Supporting File: advs75083‐sup‐0001‐SuppMat.docx. [file ADVS-13-e23984-s001.docx]

**INB³P: A Multi-Modal and Interpretable Co-Attention Framework Integrating Property-Aware Explanations and Memory-Bank Contrastive Fusion for Blood–Brain Barrier Penetrating Peptide Discovery**

Jingwei Lv^1^, Qianyang Wu^1^, Jian Liu^1^, Binlu Yang^1^, Yuanhao Li^1^, Junlin Xu^2^, Yajie Meng^3^, Leyi Wei^4^, Zilong Zhang^1^, Quan Zou^5^, Xiulai Li^1,^ *, Feifei Cui^1^^,^ *

*^1^School of Computer Science and Technology, Hainan University, Haikou, 570228, China*

*^2^School of Computer Science and Technology, Wuhan University of Science and Technology, Wuhan**, 430081, Hubei, China*

*^3^School of Computer Science and Artificial Intelligence, Wuhan Textile University, Wuhan, 430200, Hubei, China*

*^4^Centre for Artificial Intelligence driven Drug Discovery, Faculty of Applied Science, Macao Polytechnic University, Macao SAR, 999078, China*

*^5^Institute of Fundamental and Frontier Sciences, University of Electronic Science and Technology of China, Chengdu, 610054, China*

*** **Corresponding author:**

Xiulai Li ([lixiulai01@hainanu.edu.cn](mailto:lixiulai01@hainanu.edu.cn)), Feifei Cui ([feifeicui@hainanu.edu.cn](mailto:feifeicui@hainanu.edu.cn))

**Supplementary Note S1. Supplementary Architecture-Level Ablation of Modality Contribution and Fusion Strategy**

To further evaluate modality contribution and fusion effectiveness in INB³P, we conducted supplementary architecture-level ablation experiments comparing five variants: sequence-only, structure-only, concatenation-based fusion, cross-/co-attention-based fusion, and the full model. Performance was assessed by cross-validation using AUC, MCC, ACC, sensitivity (Sn), specificity (Sp), and training time per epoch.

The unimodal variants were clearly less balanced than the multimodal configurations. The sequence-only model achieved relatively high ACC and very high specificity, but substantially lower sensitivity, indicating a strong bias toward the majority class. The structure-only model improved sensitivity relative to the sequence-only setting, but still remained inferior to multimodal variants in overall balanced performance. These observations suggest that both modalities contain useful signal, but neither alone is sufficient for robust BBBPP prediction under the current benchmark.

By contrast, the multimodal variants consistently improved AUC and positive-class detection. Comparing fusion strategies, the concatenation-based model already captured part of the multimodal gain, but the co-attention-based model provided stronger overall discrimination, indicating that explicit sequence–structure interaction modeling is more effective than direct feature aggregation. In addition, compared with simple concatenation, the bidirectional co-attention mechanism offers a clearer interpretability pathway by producing directional attention values from both the sequence and structure sides, thereby allowing residue importance to be examined from complementary representational perspectives.

Interestingly, the co-attention-based variant performed similarly to the full model in this supplementary comparison, whereas the full model incurred the highest computational cost. This suggests that the principal gain may arise from explicit cross-modal interaction itself, while additional architectural complexity does not necessarily translate into further improvement in this setting. Overall, these results reinforce the conclusion that the performance of INB³P depends not only on multimodal input availability, but also on the design of the interaction mechanism used to integrate those inputs.

The results are reported in Supplementary Table SN1 and the detailed results are reported in Supplementary Table SN2.

**Table SN1.** Architecture-level ablation of modality contribution and fusion strategy in INB³P.

| **Model Variant** | **AUC** | **MCC** | **ACC** | **Sn** | **Sp** | **Time* (s/epoch)** |
| --- | --- | --- | --- | --- | --- | --- |
| seq_only | 0.737± 0.073 | 0.536±0.030 | 0.967±0.002 | 0.377±0.027 | 0.996±0.001 | 31.09 ± 2.75 |
| struct_only | 0.830±0.049 | 0.298±0.067 | 0.903±0.008 | 0.501±0.121 | 0.923±0.011 | 33.49 ± 22.67 |
| concat | 0.877±0.022 | 0.347±0.020 | 0.851±0.012 | 0.775±0.044 | 0.855±0.013 | 45.96 ± 3.01 |
| cross_attn | 0.883±0.023 | 0.348±0.022 | 0.844±0.012 | 0.796±0.045 | 0.847±0.013 | 47.46 ± 3.35 |
| full | 0.846±0.051 | 0.337±0.033 | 0.836±0.023 | 0.793±0.015 | 0.838±0.023 | 75.75 ± 6.16 |

*Time denotes the per-epoch runtime (seconds), jointly accounting for Stage-1 (35 epochs) and Stage-2 (15 epochs). For each fold, we first sum the per-epoch means of modules A and B and approximate the combined SD as $s_{\text{tot}}=\sqrt{s_{A}^{2}+s_{B}^{2}}$; we then compute a pooled summary weighted by the stage epoch counts (35 and 15), and finally report the 5-fold mean ± pooled SD.

**Table SN2.** Fold-wise cross-validation results for the architecture-level ablation of modality contribution and fusion strategy in INB³P.

| **Model Variant** | **Fold** | **ACC** | **Sn** | **Sp** | **MCC** | **AUC** |
| --- | --- | --- | --- | --- | --- | --- |
| seq_only | 1 | 0.969 | 0.409 | 0.996 | 0.566 | 0.779 |
|  | 2 | 0.967 | 0.348 | 0.996 | 0.522 | 0.674 |
|  | 3 | 0.969 | 0.394 | 0.996 | 0.562 | 0.747 |
|  | 4 | 0.964 | 0.348 | 0.994 | 0.494 | 0.831 |
|  | 5 | 0.967 | 0.385 | 0.995 | 0.534 | 0.656 |
|  |  |  |  |  |  |  |
| struct_only | 1 | 0.906 | 0.545 | 0.923 | 0.330 | 0.857 |
|  | 2 | 0.896 | 0.545 | 0.913 | 0.309 | 0.853 |
|  | 3 | 0.901 | 0.652 | 0.913 | 0.374 | 0.859 |
|  | 4 | 0.916 | 0.424 | 0.939 | 0.285 | 0.837 |
|  | 5 | 0.898 | 0.338 | 0.924 | 0.193 | 0.743 |
|  |  |  |  |  |  |  |
| concat | 1 | 0.847 | 0.803 | 0.849 | 0.355 | 0.869 |
|  | 2 | 0.854 | 0.818 | 0.855 | 0.371 | 0.895 |
|  | 3 | 0.835 | 0.758 | 0.839 | 0.319 | 0.848 |
|  | 4 | 0.852 | 0.788 | 0.855 | 0.354 | 0.902 |
|  | 5 | 0.868 | 0.708 | 0.875 | 0.338 | 0.871 |
|  |  |  |  |  |  |  |
| cross_attn | 1 | 0.849 | 0.803 | 0.851 | 0.357 | 0.858 |
|  | 2 | 0.830 | 0.848 | 0.829 | 0.353 | 0.911 |
|  | 3 | 0.836 | 0.803 | 0.838 | 0.341 | 0.879 |
|  | 4 | 0.860 | 0.803 | 0.863 | 0.373 | 0.902 |
|  | 5 | 0.846 | 0.723 | 0.852 | 0.315 | 0.867 |
|  |  |  |  |  |  |  |
|  | 1 | 0.862 | 0.803 | 0.864 | 0.375 | 0.869 |
|  | 2 | 0.844 | 0.803 | 0.846 | 0.351 | 0.904 |
| full | 3 | 0.840 | 0.788 | 0.842 | 0.339 | 0.825 |
|  | 4 | 0.833 | 0.803 | 0.834 | 0.337 | 0.863 |
|  | 5 | 0.800 | 0.769 | 0.801 | 0.284 | 0.771 |

**Supplementary Note S2. Label Provenance and Benchmark Construction**

The benchmark dataset used in this study was adopted directly from the DeepB3P benchmark curated by Tang et al. [1], and no re-splitting, post hoc relabeling, or additional manual curation was performed in the present work. This design choice was made to preserve strict comparability with previously reported BBBPP predictors evaluated under the same benchmark setting.

**Provenance of positive labels**

The positive class in the DeepB3P benchmark corresponds to blood–brain barrier-penetrating peptides (BBBPPs). As described by Tang et al. [1], these positive samples were obtained by extending the earlier BBPpredict dataset [2]. In turn, the BBPpredict positive dataset was derived from B3Pdb [3], a literature-curated database of BBB-crossing peptides. B3Pdb was constructed through manual curation of relevant research articles and patents retrieved from PubMed and related sources using combinations of blood–brain barrier- and peptide-related search terms. The database records information associated with BBB-penetrating peptides, including sequence, peptide length, chemical modifications, in vitro/in vivo activity, model systems, uptake efficiency, and therapeutic properties, where available. Accordingly, the positive labels used in the present benchmark ultimately originate from previously reported BBB-related peptide evidence collected from heterogeneous literature sources rather than from a single experimental platform.

**Provenance of negative labels**

The negative class in the Tang et al. benchmark corresponds to non-BBBPP controls. According to the benchmark construction protocol, these negatives were generated by applying the BBPpredict UniProt filtering strategy. Specifically, UniProt sequences were filtered using keyword-based exclusion rules to remove entries associated with BBB/brain-related and membrane/transport-related concepts, including BBB, brain, brain peptides, permeation, permeability, venom, toxin, transmembrane, transport, transfer, membrane, neuro, and hemolysis. Additional quality-control constraints were then applied, including retention of reviewed sequences only, restriction of peptide length to 5–50 amino acids, and exclusion of sequences containing ambiguous residues. Finally, redundancy reduction was performed using CD-HIT at a 40% identity threshold. The independent testing dataset reused the positive and negative testing sets released with BBPpredict, following Tang et al.

**Final benchmark composition**

Using this construction pipeline, the final DeepB3P benchmark comprised 329 BBBPPs and 6,851 non-BBBPPs for model training, together with 99 BBBPPs and 99 non-BBBPPs for independent testing. All peptide sequences fell within the length range of 5–50 amino acids. These train/test partitions were adopted as released in the benchmark and used unchanged throughout the present study.

**Inter-platform variability and interpretational scope**

An important consideration is that the positive labels in this benchmark were aggregated from heterogeneous source studies, including literature-curated peptide resources that contain evidence from multiple experimental contexts rather than a single homogeneous assay platform. Although B3Pdb records information related to in vitro/in vivo activities and model systems where available, the released DeepB3P benchmark does not provide a unified per-sample harmonization scheme for experimental platform effects, nor does it explicitly support platform-stratified evaluation within the published benchmark split. As a result, inter-platform variability was not explicitly corrected in the present study.

For this reason, the results reported in this manuscript should be interpreted primarily as comparative performance under an established literature benchmark, rather than as a definitive assessment of cross-platform robustness. Addressing platform heterogeneity more explicitly would require future reconstruction of a platform-annotated benchmark with harmonized metadata and sufficiently large sample sizes across assay types to support stratified training and evaluation.

**Rationale for using the benchmark as released**

We intentionally adopted the DeepB3P benchmark as released because our primary objective was to evaluate INB³P under the same conditions used by prior BBBPP predictors, thereby enabling direct and fair comparison. Reconstructing labels, redefining negatives, or re-splitting the data would have introduced an additional source of variability and would have reduced comparability with the published literature. Accordingly, all conclusions in the present study should be understood within the scope of this benchmark setting.

**Supplementary Note S3. Rationale for the Two-Stage Optimization Curriculum**

The two-stage optimization schedule in INB³P was introduced to separate two partially competing learning objectives: (i) shaping a stable multimodal representation space through augmentation and contrastive alignment, and (ii) refining a discriminative decision boundary for the final imbalanced BBBPP classification task. In Stage-1, the optimization emphasizes representation learning, encouraging sequence and structure views to align under supervised and multimodal contrastive objectives while remaining robust to augmentation. In Stage-2, the optimization shifts toward classification-oriented refinement through focal and differentiable MCC losses, which are more directly tied to the final decision boundary and class-imbalance handling.

We did not optimize all objectives jointly from the beginning because their functional roles are different and may interfere during early training. Contrastive objectives primarily act on the geometry of the embedding space, whereas classification losses act more directly on label-separating decision surfaces. Prior work has noted that contrastive and classification objectives can behave as partially competing optimization targets rather than perfectly aligned ones, especially when optimized simultaneously under limited-data conditions. [4] In our setting, this issue is further amplified by severe class imbalance and augmentation-induced perturbations. If the classification-oriented losses dominate too early, the model may converge prematurely to label-driven shortcuts before cross-modal structure and representation alignment have stabilized.

The mild curriculum therefore serves two purposes. First, it improves optimization stability by allowing the model to establish a more coherent joint sequence–structure representation before the final classifier is strongly emphasized. Second, it improves convergence behavior by reducing interference between early representation shaping and later decision-boundary refinement. Empirically, this design produced more stable training and better final performance than directly applying all objectives from the start, and was therefore retained in the final framework.

Accordingly, the two-stage design should not be interpreted as introducing two unrelated training procedures, but rather as a controlled optimization schedule in which Stage-1 focuses on representation organization and cross-modal alignment, while Stage-2 focuses on prediction-oriented calibration under class imbalance.

**Supplementary Note S4. Rationale for Selecting ESM-2 as the Sequence Encoder**

Protein language models (PLMs) were introduced into INB³P to provide transferable sequence representations under extreme data scarcity, where labeled BBBPP samples are limited and conventional task-specific encoders are more prone to overfitting. In this setting, a pretrained protein encoder offers a practical way to inject large-scale sequence knowledge into downstream supervised peptide prediction.

In the present study, ESM-2 was selected as the sequence encoder because our framework was designed not only for benchmark evaluation, but also for reproducible local execution, user-side extensibility, and web-server deployment. Accordingly, we prioritized a backbone with an established practical workflow for supervised peptide modeling, including stable local inference, straightforward integration into downstream training pipelines, and broad accessibility for users with different computational resources.

This choice should not be interpreted as implying that newer protein foundation models, such as ESM-C or ESM3, are less capable. On the contrary, these newer models are highly promising and may offer stronger representations in some settings. However, the goal of the present study was to evaluate the contribution of the proposed augmentation, multimodal fusion, and interpretability framework under a controlled and reproducible backbone configuration, rather than to optimize performance solely through adopting the newest available encoder. For this reason, we chose ESM-2 as a strong and practical baseline backbone for the current version of INB³P.

At the same time, systematic benchmarking of INB³P with newer protein foundation models will be an important direction for future work. In particular, evaluating ESM-C- or ESM3-based variants under the same small-sample supervised peptide prediction setting may help determine whether newer backbone models further improve robustness, generalization, or biological interpretability.

**Table S1.** Ablation analysis on the depth of the cross-modal fusion module.

| **Fusion layer** | **Fold** | **ACC** | **Sn** | **Sp** | **MCC** | **AUC** | **AP** |
| --- | --- | --- | --- | --- | --- | --- | --- |
| 1 | 1 | 0.795 | 0.864 | 0.791 | 0.322 | 0.873 | 0.353 |
|  | 2 | 0.860 | 0.758 | 0.865 | 0.352 | 0.891 | 0.233 |
|  | 3 | 0.817 | 0.773 | 0.819 | 0.305 | 0.807 | 0.298 |
|  | 4 | 0.780 | 0.833 | 0.777 | 0.295 | 0.853 | 0.179 |
|  | 5 | 0.842 | 0.723 | 0.847 | 0.310 | 0.809 | 0.184 |
|  |  |  |  |  |  |  |  |
| 2 | 1 | 0.848 | 0.848 | 0.848 | 0.377 | 0.864 | 0.215 |
|  | 2 | 0.861 | 0.833 | 0.862 | 0.388 | 0.901 | 0.228 |
|  | 3 | 0.843 | 0.758 | 0.847 | 0.330 | 0.867 | 0.396 |
|  | 4 | 0.848 | 0.773 | 0.852 | 0.343 | 0.881 | 0.234 |
|  | 5 | 0.845 | 0.708 | 0.852 | 0.307 | 0.793 | 0.185 |
|  |  |  |  |  |  |  |  |
| 3 | 1 | 0.046 | 1.000 | 0.000 | 0.000 | 0.175 | 0.037 |
|  | 2 | 0.046 | 1.000 | 0.000 | 0.000 | 0.501 | 0.046 |
|  | 3 | 0.046 | 1.000 | 0.000 | 0.000 | 0.822 | 0.144 |
|  | 4 | 0.046 | 1.000 | 0.000 | 0.000 | 0.900 | 0.494 |
|  | 5 | 0.916 | 0.662 | 0.928 | 0.412 | 0.833 | 0.239 |
|  |  |  |  |  |  |  |  |
| 4 | 1 | 0.045929 | 1 | 0 | 0 | 0.486528 | 0.044946 |
|  | 2 | 0.045961 | 1 | 0 | 0 | 0.623828 | 0.061477 |
|  | 3 | 0.046657 | 1 | 0.00073 | 0.005794 | 0.831724 | 0.584055 |
|  | 4 | 0.919916 | 0.742424 | 0.928467 | 0.463446 | 0.852499 | 0.315598 |
|  | 5 | 0.045296 | 1 | 0 | 0 | 0.5 | 0.045296 |

**Table S2.** Ablation study on the scaling of the ESM-2 sequence encoder.

| **ESM-2 version** | **Fold** | **ACC** | **Sn** | **Sp** | **MCC** | **AUC** | **AP** |
| --- | --- | --- | --- | --- | --- | --- | --- |
| 8M | 1 | 0.811 | 0.879 | 0.807 | 0.345 | 0.920 | 0.580 |
|  | 2 | 0.808 | 0.803 | 0.808 | 0.309 | 0.917 | 0.439 |
|  | 3 | 0.852 | 0.758 | 0.857 | 0.342 | 0.891 | 0.479 |
|  | 4 | 0.841 | 0.727 | 0.847 | 0.313 | 0.902 | 0.431 |
|  | 5 | 0.847 | 0.677 | 0.855 | 0.295 | 0.895 | 0.417 |
|  |  |  |  |  |  |  |  |
| 35M | 1 | 0.827 | 0.833 | 0.826 | 0.343 | 0.906 | 0.373 |
|  | 2 | 0.844 | 0.788 | 0.847 | 0.344 | 0.904 | 0.248 |
|  | 3 | 0.873 | 0.818 | 0.875 | 0.399 | 0.922 | 0.524 |
|  | 4 | 0.752 | 0.864 | 0.747 | 0.284 | 0.897 | 0.281 |
|  | 5 | 0.843 | 0.738 | 0.847 | 0.318 | 0.890 | 0.249 |
|  |  |  |  |  |  |  |  |
| 150M | 1 | 0.802 | 0.848 | 0.799 | 0.322 | 0.861 | 0.221 |
|  | 2 | 0.874 | 0.788 | 0.878 | 0.388 | 0.896 | 0.337 |
|  | 3 | 0.872 | 0.773 | 0.877 | 0.378 | 0.929 | 0.458 |
|  | 4 | 0.878 | 0.818 | 0.881 | 0.409 | 0.913 | 0.305 |
|  | 5 | 0.838 | 0.785 | 0.840 | 0.332 | 0.889 | 0.248 |
|  |  |  |  |  |  |  |  |
| 650M | 1 | 0.818 | 0.864 | 0.816 | 0.346 | 0.888 | 0.222 |
|  | 2 | 0.859 | 0.773 | 0.864 | 0.358 | 0.857 | 0.229 |
|  | 3 | 0.823 | 0.788 | 0.825 | 0.319 | 0.777 | 0.183 |
|  | 4 | 0.829 | 0.833 | 0.828 | 0.345 | 0.904 | 0.318 |
|  | 5 | 0.864 | 0.723 | 0.871 | 0.340 | 0.875 | 0.216 |

**Table S3.** Ablation study on contrastive learning objectives during the representation learning stage.

| **Loss function** | **Fold** | **ACC** | **Sn** | **Sp** | **MCC** | **AUC** | **AP** |
| --- | --- | --- | --- | --- | --- | --- | --- |
| InfoNCE | 1 | 0.812 | 0.833 | 0.811 | 0.326 | 0.910 | 0.409 |
|  | 2 | 0.796 | 0.909 | 0.791 | 0.342 | 0.918 | 0.378 |
|  | 3 | 0.823 | 0.773 | 0.826 | 0.312 | 0.893 | 0.469 |
|  | 4 | 0.767 | 0.894 | 0.761 | 0.309 | 0.916 | 0.468 |
|  | 5 | 0.814 | 0.785 | 0.815 | 0.305 | 0.892 | 0.371 |
|  |  |  |  |  |  |  |  |
| SCL(seq) + SCL(pdb) | 1 | 0.841 | 0.848 | 0.840 | 0.367 | 0.898 | 0.460 |
|  | 2 | 0.858 | 0.818 | 0.860 | 0.377 | 0.892 | 0.252 |
|  | 3 | 0.866 | 0.773 | 0.870 | 0.368 | 0.832 | 0.327 |
|  | 4 | 0.846 | 0.803 | 0.848 | 0.354 | 0.877 | 0.225 |
|  | 5 | 0.849 | 0.692 | 0.856 | 0.305 | 0.835 | 0.182 |
|  |  |  |  |  |  |  |  |
| InfoNCE + SCL(seq) + SCL(pdb) | 1 | 0.795 | 0.864 | 0.791 | 0.322 | 0.873 | 0.353 |
|  | 2 | 0.860 | 0.758 | 0.865 | 0.352 | 0.891 | 0.233 |
|  | 3 | 0.817 | 0.773 | 0.819 | 0.305 | 0.807 | 0.298 |
|  | 4 | 0.780 | 0.833 | 0.777 | 0.295 | 0.853 | 0.179 |
|  | 5 | 0.842 | 0.723 | 0.847 | 0.310 | 0.809 | 0.184 |

**Table S4.** Tuning the weight balance between Focal Loss and Stable-MCC for imbalance-robust classification.

| **short_key** | **Fold** | **ACC** | **Sn** | **Sp** | **MCC** | **AUC** | **AP** |
| --- | --- | --- | --- | --- | --- | --- | --- |
| wf0.20 | 1 | 0.85595 | 0.833333 | 0.857039 | 0.380623 | 0.880733 | 0.238728 |
|  | 2 | 0.858635 | 0.772727 | 0.862774 | 0.357275 | 0.916838 | 0.294664 |
|  | 3 | 0.853064 | 0.787879 | 0.856204 | 0.356245 | 0.854921 | 0.207841 |
|  | 4 | 0.849582 | 0.787879 | 0.852555 | 0.351464 | 0.892684 | 0.245955 |
|  | 5 | 0.865505 | 0.707692 | 0.872993 | 0.335165 | 0.868276 | 0.19866 |
|  |  |  |  |  |  |  |  |
| wf0.60 | 1 | 0.851079 | 0.80303 | 0.853392 | 0.360217 | 0.890928 | 0.610847 |
|  | 2 | 0.862117 | 0.818182 | 0.864234 | 0.38301 | 0.913531 | 0.282861 |
|  | 3 | 0.855153 | 0.757576 | 0.859854 | 0.34539 | 0.847744 | 0.487298 |
|  | 4 | 0.851671 | 0.80303 | 0.854015 | 0.361139 | 0.879164 | 0.238806 |
|  | 5 | 0.836237 | 0.692308 | 0.843066 | 0.289047 | 0.856552 | 0.191888 |
|  |  |  |  |  |  |  |  |
| wf0.70 | 1 | 0.841336 | 0.833333 | 0.841721 | 0.36073 | 0.889966 | 0.409295 |
|  | 2 | 0.824513 | 0.833333 | 0.824088 | 0.340312 | 0.904197 | 0.586292 |
|  | 3 | 0.853064 | 0.757576 | 0.857664 | 0.342482 | 0.893055 | 0.299311 |
|  | 4 | 0.847493 | 0.80303 | 0.849635 | 0.355455 | 0.896522 | 0.23981 |
|  | 5 | 0.839721 | 0.723077 | 0.845255 | 0.307302 | 0.891606 | 0.401748 |
|  |  |  |  |  |  |  |  |
| wf0.80 | 1 | 0.848295 | 0.818182 | 0.849745 | 0.363199 | 0.895188 | 0.529333 |
|  | 2 | 0.848189 | 0.772727 | 0.851825 | 0.342748 | 0.923689 | 0.480917 |
|  | 3 | 0.821727 | 0.818182 | 0.821898 | 0.330512 | 0.87526 | 0.383361 |
|  | 4 | 0.839833 | 0.787879 | 0.842336 | 0.338713 | 0.88069 | 0.211937 |
|  | 5 | 0.854355 | 0.723077 | 0.860584 | 0.326319 | 0.874615 | 0.213317 |
|  |  |  |  |  |  |  |  |
| wf0.30 | 1 | 0.861517 | 0.80303 | 0.864333 | 0.375168 | 0.913324 | 0.471773 |
|  | 2 | 0.847493 | 0.787879 | 0.850365 | 0.348655 | 0.90245 | 0.248034 |
|  | 3 | 0.834958 | 0.787879 | 0.837226 | 0.332662 | 0.850697 | 0.194374 |
|  | 4 | 0.791086 | 0.833333 | 0.789051 | 0.305352 | 0.876388 | 0.187373 |
|  | 5 | 0.852265 | 0.753846 | 0.856934 | 0.337556 | 0.870157 | 0.196812 |
|  |  |  |  |  |  |  |  |
| wf0.40 | 1 | 0.832985 | 0.833333 | 0.832969 | 0.350258 | 0.86152 | 0.232719 |
|  | 2 | 0.843315 | 0.80303 | 0.845255 | 0.349943 | 0.895211 | 0.243611 |
|  | 3 | 0.837047 | 0.772727 | 0.840146 | 0.328452 | 0.884301 | 0.309718 |
|  | 4 | 0.835655 | 0.787879 | 0.837956 | 0.333514 | 0.873955 | 0.29442 |
|  | 5 | 0.857143 | 0.692308 | 0.864964 | 0.315883 | 0.871331 | 0.217524 |
|  |  |  |  |  |  |  |  |
| wf0.50 | 1 | 0.862213 | 0.818182 | 0.864333 | 0.383045 | 0.925955 | 0.568412 |
|  | 2 | 0.839833 | 0.80303 | 0.841606 | 0.345474 | 0.888885 | 0.200661 |
|  | 3 | 0.809889 | 0.818182 | 0.809489 | 0.317619 | 0.86127 | 0.263084 |
|  | 4 | 0.857939 | 0.742424 | 0.863504 | 0.342379 | 0.872163 | 0.225723 |
|  | 5 | 0.83554 | 0.676923 | 0.843066 | 0.281162 | 0.813773 | 0.265166 |
|  |  |  |  |  |  |  |  |
| wf0.10 | 1 | 0.837161 | 0.848485 | 0.836616 | 0.362076 | 0.905527 | 0.263381 |
|  | 2 | 0.839136 | 0.863636 | 0.837956 | 0.37132 | 0.907344 | 0.233452 |
|  | 3 | 0.821727 | 0.772727 | 0.824088 | 0.310504 | 0.794321 | 0.195377 |
|  | 4 | 0.811281 | 0.80303 | 0.811679 | 0.312497 | 0.84134 | 0.175446 |
|  | 5 | 0.852265 | 0.661538 | 0.861314 | 0.294826 | 0.854318 | 0.18539 |
|  |  |  |  |  |  |  |  |
| wf0.90 | 1 | 0.8135 | 0.848485 | 0.811816 | 0.334486 | 0.895315 | 0.47588 |
|  | 2 | 0.85376 | 0.80303 | 0.856204 | 0.364049 | 0.892186 | 0.217673 |
|  | 3 | 0.840529 | 0.742424 | 0.845255 | 0.319105 | 0.887696 | 0.426737 |
|  | 4 | 0.833565 | 0.80303 | 0.835036 | 0.337699 | 0.885352 | 0.238348 |
|  | 5 | 0.841115 | 0.692308 | 0.848175 | 0.294937 | 0.87219 | 0.272739 |
|  |  |  |  |  |  |  |  |
| wf1.00 | 1 | 0.828114 | 0.80303 | 0.829322 | 0.3311 | 0.897144 | 0.428125 |
|  | 2 | 0.834958 | 0.80303 | 0.836496 | 0.339398 | 0.874724 | 0.237825 |
|  | 3 | 0.809192 | 0.742424 | 0.812409 | 0.283723 | 0.831298 | 0.587833 |
|  | 4 | 0.841226 | 0.80303 | 0.843066 | 0.347249 | 0.892723 | 0.41083 |
|  | 5 | 0.848084 | 0.723077 | 0.854015 | 0.317911 | 0.881308 | 0.469171 |

**Table S5.** General Setting.

| **Parameter** | **Value (runs)** | **Default** | **Description** |
| --- | --- | --- | --- |
| --train_csv | /home/Euclid/BBBPP/data/trainV1.csv | — (required) | Training data CSV; must have sequence, label (optional pdb_dir). |
| --test_csv | /home/Euclid/BBBPP/data/testV1.csv | — (required) | Held-out test data CSV. |
| --output_dir | ./outputs | ./outputs | Where logs, figures, and checkpoints are written. |
| --hid | 1024 | 1024 | Model hidden size for fusion/heads. |
| --batch | 64 | 64 | Effective batch size for DataLoader (stratified batches). |
| --seed | 45 | 45 | Global RNG seed for reproducibility. |
| --dropout | 0.5 | 0.5 | Base dropout used in modules (overridden in Stage-2 if set). |
| --deterministic | Yes | No | Enables torch.use_deterministic_algorithms(True, warn_only=True); combined with CUBLAS_WORKSPACE_CONFIG=":16:8" for stricter reproducibility. |
| --val_split_ratio | 0.0 | 0.0 | When >0, split train into train/val for early stopping & LR scheduler. You trained with no explicit val split (train on all). |

**Table S6.** Stage-1 (Representation learning).

| **Parameter** | **Value (runs)** | **Default** | **Description** |
| --- | --- | --- | --- |
| --stage1_epochs | 35 | 35 | Number of Stage-1 epochs. |
| --lr_stage1 | 2e-5 | 2e-5 | AdamW LR for all trainable params in Stage-1. |
| --stage1_cls_loss_weight | 0.5 | 0.5 | Curriculum: mixes supervised loss into Stage-1 (scales with epoch progression). |
| --freeze_ratio_esm | 0.7 | 0.7 | Freeze the **first 70%** of ESM layers during Stage-1 to stabilize training. |

**Table S7.** Stage-2 (Fine-tuning).

| **Parameter** | **Value (runs)** | **Default** | **Description** |
| --- | --- | --- | --- |
| --stage2_epochs | 15 | 15 | Number of Stage-2 epochs. |
| --lr_stage2_head | 2e-4 | 2e-4 | LR for classifier/head parameters. |
| --lr_stage2_encoder | 1e-6 | 1e-6 | LR for encoder (ESM + GNN/backbone). |
| --stage2_dropout | 0.5 | 0.5 | Resets dropout in modules (and MHA) for Stage-2. |
| --stage2_unfreeze_ratio | 0.8 | None | Unfreezes ESM layers from int(0.8 * num_layers) onward ⇒ last 20% are trainable in Stage-2. |
| Early-stopping & LR scheduler | (inactive) | (on when val) | ReduceLROnPlateau(+ES) activate only if val_split_ratio>0. You trained without validation, so the final epoch checkpoint is used for test. |

**Table S8.** Augmentation & Folding.

| **Parameter** | **Value (runs)** | **Default** | **Description** |
| --- | --- | --- | --- |
| --enable_esmfold | Yes | No | Enables **on-the-fly ESMFold** for mutated or missing-PDB sequences (heavy). |
| --esmfold_cache_dir | ./esmfold_cache | None | Cache dir for folded PDB text; avoids refolding identical sequences. |
| --aug_prob | 0.5 | 0.5 | Per-sample probability to attempt PCGM/BCGM mutation in Stage-1 (positive class only). |
| --mutation_prob | 0.3 | 0.3 | Per-position mutation probability when augmentation is active. |
| --max_mutation_fraction | 0.2 | 0.2 | Caps the fraction of mutated residues per sequence. |
| --aug_warmup_epochs | 3 | 0 | Linearly warms up aug_prob over first 3 epochs to reduce early instability. |
| --enable_stage2_augmentation | Yes | No | Also apply augmentation in Stage-2. |
| --stage2_aug_prob | 1.0 | 1.0 | Stage-2 augmentation probability. |
| --stage2_mutation_prob | 0.25 | 0.25 | Stage-2 per-position mutation probability. |
| --stage2_max_mutation_fraction | 0.25 | 0.25 | Stage-2 cap on mutated fraction. |

**Table S9.** Losses, banks, and contrastive setup.

| **Parameter** | **Value (runs)** | **Default** | **Description** |
| --- | --- | --- | --- |
| --bank_size | 2048 | 2048 | Size of modality-specific memory banks for InfoNCE negatives. |
| --k_neg_infonce | 64 | 64 | Number of sampled negatives per anchor in InfoNCE. |
| --lambda_contrastive | 1.0 | 1.0 | Weight for InfoNCE in Stage-1 (before curriculum mixing). |
| --lambda_scl_seq | 2.0 | 2.0 | Weight for supervised contrastive loss (sequence branch). |
| --lambda_scl_pdb | 2.0 | 2.0 | Weight for supervised contrastive loss (structure/graph branch). |
| --scl_embedding_dim | 128 | 128 | Projection head dim for SCL embeddings. |
| --scl_temperature | 0.1 | 0.1 | Temperature for SCL. |
| --gamma_focal | 2.0 | 2.0 | Focusing parameter γ in focal loss. |
| --w_focal | 0.2 | 0.2 | Weight of focal loss in supervised objective (Stage-1 mix & Stage-2). |
| --w_mcc | 0.8 | 0.8 | Weight of stable MCC loss in supervised objective. |
| --label_smoothing | 0.1 | 0.1 | Label smoothing applied in classification losses (incl. MCC variant). |

**Table S10.** ESM channel handling & memory.

| **Parameter** | **Value (runs)** | **Default** | **Description** |
| --- | --- | --- | --- |
| --normalize_esm_channels | Yes | No | Z-score normalize per-channel ESM token embeddings using provided stats. |
| --esm_channel_stats_path | ./esm2_650M_channel_stats.npz | None | Path to precomputed mean/std for channels; required when normalization is enabled. |
| --esm_grad_ckpt | Yes | No | Enables **gradient checkpointing** inside ESM to reduce memory at a small compute cost. |

**Table S11.** PCGM mutation sampler.

| **Parameter** | **Value (runs)** | **Default** | **Description** |
| --- | --- | --- | --- |
| --w_blosum | 0.5 | 0.5 | Mixture weight for min-max normalized **BLOSUM62** row (evolutionary plausibility). |
| --w_biochem | 0.5 | 0.5 | Mixture weight for **physicochemical similarity** kernel. |
| --softmax_temp | 0.3 | 0.3 | Row-wise softmax temperature for mutation distribution (lower ⇒ peakier, fewer radical substitutions). |

**Table S12.** Fusion & architecture toggles.

| **Parameter** | **Value (runs)** | **Default** | **Description** |
| --- | --- | --- | --- |
| --num_fusion_layers | 1 | 1 | Number of cross-modal fusion layers in BP_INFP. |
| PDB_NODE_FEATURE_DIM | (package const) | — | Dimension of node features from PDB graph encoder (imported from package). |

**Table S13.** Computing environment used for training and evaluation of INB³P.

| **Component** | **Specification** |
| --- | --- |
| Operating system | CentOS Stream 9 (64-bit) |
| CPU | AMD Ryzen 9 7950X, 16 cores / 32 threads |
| GPU | NVIDIA GeForce RTX 4090, 24 GB GDDR6X |
| System memory (RAM) | 61.8 GiB |
| Storage | 10 TB |
| NVIDIA driver / CUDA | Driver 535.274.02, CUDA 12.2 (runtime) |
| Python | 3.8.19 |
| PyTorch / cuDNN | PyTorch 1.13.1 (CUDA 11.6, cuDNN 8.3.2) |
| PyTorch Geometric | 2.5.2 |
| ESM / ESMFold | \|  \| \| --- \|  \| fair-esm 2.0.0 (ESMFold esmfold_v1 pretrained model) \| \| --- \| |

**Table S14.** Summary of physicochemical properties and their corresponding references.

| Properties name | Reference |
| --- | --- |
| hydropathy_KyteDoolittle | [5] |
| molecular_weight_Da | [6] |
| isoelectric_point_pI | [7] |
| net_charge_at_pH7 | [8] |
| aromatic_flag | [9] |
| sidechain_SASA_A2 | [10] |
| molar_refractivity_scaled | [11] |
| WimleyWhite_interfacial_hydrophobicity_kcal_per_mol | [12] |
| TPSA_A2 | [13] |

**Supplementary Algorithm S1:** BCGM – Biochemistry-Guided Mutagenesis Augmentation.

| Inputs:  S = (a1,...,aL) # amino-acid sequence  A = canonical 20 AA  B(x,y) # BLOSUM score  p_hat(x) ∈ R^d # z-scored property vector for residue x  Hyperparams: p_aug, p_mut, rho_max, α, β, τ, T  FLAGS: forbid_identity (bool), log_path (optional)  1: if Bernoulli(p_aug) == 0 then  2: return S # no augmentation  3: end if  4: # Precompute substitution distribution per residue type  5: for each x in A do  6: for each y in A do  7: K_prop[x,y] ← exp( - \|\|p_hat(x) - p_hat(y)\|\|_2^2 / (2 τ^2) )  8: K_blo [x,y] ← exp( β * B(x,y) )  9: end for  10: # Normalize K_blo row-wise  11: K_blo[x,*] ← K_blo[x,*] / sum_y K_blo[x,y]  12: K_fuse[x,*] ← (K_blo[x,*])^α ⊙ (K_prop[x,*])^(1-α)  13: if forbid_identity then K_fuse[x,x] ← 0 end if  14: # Temperatured softmax  15: P_sub[x,*] ← (K_fuse[x,*])^(1/T) / sum_y (K_fuse[x,y])^(1/T)  16: end for  17:  18: # Select positions to mutate  19: M ← {} # set of indices to mutate  20: for i = 1..L do  21: if Bernoulli(p_mut) == 1 then add i to M end if  22: end for  23: if \|M\| > floor(rho_max * L) then  24: M ← random_subset(M, floor(rho_max * L))  25: end if  26:  27: # Apply substitutions  28: S_tilde ← S  29: for each i in M do  30: x ← a_i  31: sample y ~ Categorical( P_sub[x,*] )  32: S_tilde[i] ← y  33: if log_path then  34: Δp ← p_hat(y) - p_hat(x)  35: append_log(log_path, i, x, y, Δp)  36: end if  37: end for  38: return S_tilde |
| --- |

**Supplementary Algorithm S2:** STRATIFIED-BATCH-SAMPLER – Stratified mini-batch sampling under class imbalance.

| Inputs:  y = (y1,...,yN) # binary labels, yi ∈ {0,1}  Hyperparams: B # batch size (number of samples per mini-batch)  shuffle # boolean, shuffle indices within each mini-batch  seed # random seed for reproducibility  1: Initialize RNG ← RandomNumberGenerator(seed)  2: POS ← { i \| yi = 1 } # indices of BBBPP (positive) samples  3: NEG ← { i \| yi = 0 } # indices of non-BBBPP (negative) samples  4: if \|POS\| == 0 or \|NEG\| == 0 then  5: # degenerate case; fall back to standard random batching  6: return RANDOM-BATCH-SAMPLER(y, B, shuffle, seed)  7: end if  8: num_batches ← ceil( N / B )  9: for t = 1..num_batches do  10: # target roughly half positives, half negatives  11: n_pos ← floor(B / 2)  12: n_neg ← B − n_pos  13: # sample positive indices  14: if \|POS\| ≥ n_pos then  15: pos_batch ← sample_without_replacement(POS, n_pos, RNG)  16: else  17: pos_batch ← sample_with_replacement(POS, n_pos, RNG)  18: end if  19: # sample negative indices  20: if \|NEG\| ≥ n_neg then  21: neg_batch ← sample_without_replacement(NEG, n_neg, RNG)  22: else  23: neg_batch ← sample_with_replacement(NEG, n_neg, RNG)  24: end if  25: batch_indices ← concatenate(pos_batch, neg_batch)  26: if shuffle == True then  27: batch_indices ← random_permutation(batch_indices, RNG)  28: end if  29: yield batch_indices # one mini-batch for this training step  30: end for  31: return |
| --- |

**Supplementary Algorithm S3:** Stable-MCC loss – Differentiable MCC surrogate for imbalanced classification

| Inputs:  z = (z1,...,zN) # logits (real-valued scores)  y = (y1,...,yN) # binary labels, yi ∈ {0,1}  Hyperparams: wp, wn # class weights for positives / negatives  η # label smoothing factor (0 ≤ η < 1)  ε # small constant for numerical stability  Output:  L_mcc # Stable-MCC loss (scalar)  1: # Convert logits to probabilities  2: for i = 1..N do  3: p_i ← sigmoid(z_i) # p_i ∈ (0,1)  4: end for  5: # Apply label smoothing toward 0.5  6: for i = 1..N do  7: ỹ_i ← y_i * (1 − η) + 0.5 * η # smoothed label  8: end for  9: # Compute soft confusion-matrix components with class weights  10: tp ← 0; tn ← 0; fp ← 0; fn ← 0  11: for i = 1..N do  12: tp ← tp + wp * ỹ_i * p_i # weighted soft true positives  13: fn ← fn + wp * ỹ_i * (1 − p_i) # weighted soft false negatives  14: fp ← fp + wn * (1 − ỹ_i) * p_i # weighted soft false positives  15: tn ← tn + wn * (1 − ỹ_i) * (1 − p_i) # weighted soft true negatives  16: end for  17: # Denominator terms with ε for stability  18: d1 ← tp + fp + ε  19: d2 ← tp + fn + ε  20: d3 ← tn + fp + ε  21: d4 ← tn + fn + ε  22: denom ← sqrt( d1 * d2 * d3 * d4 ) + ε  23: # Soft MCC and loss  24: numer ← tp * tn − fp * fn  25: mcc_soft ← numer / denom  26: L_mcc ← 1 − mcc_soft  27: return L_mcc |
| --- |

**References**

1. Tang Q, Chen W: **DeepB3P: A transformer-based model for identifying blood-brain barrier penetrating peptides with data augmentation using feedback GAN**. *Journal of Advanced Research* 2025, **73**:459-468.

2. Chen X, Zhang Q, Li B, Lu C, Yang S, Long J, He B, Chen H, Huang J: **BBPpredict: a web service for identifying blood-brain barrier penetrating peptides**. *Frontiers in Genetics* 2022, **13**:845747.

3. Kumar V, Patiyal S, Dhall A, Sharma N, Raghava GPS: **B3pred: A random-forest-based method for predicting and designing blood–brain barrier penetrating peptides**. *Pharmaceutics* 2021, **13**(8):1237.

4. Moukafih Y, Ghogho M, Smaili K: **Supervised contrastive learning as multi-objective optimization for fine-tuning large pre-trained language models**. In: *ICASSP 2023-2023 IEEE International Conference on Acoustics, Speech and Signal Processing (ICASSP): 2023*. IEEE: 1-5.

5. Kyte J, Doolittle RF: **A simple method for displaying the hydropathic character of a protein**. *Journal of molecular biology* 1982, **157**(1):105-132.

6. Meija J, Coplen TB, Berglund M, Brand WA, De Bièvre P, Gröning M, Holden NE, Irrgeher J, Loss RD, Walczyk T: **Atomic weights of the elements 2013 (IUPAC Technical Report)**. *Pure and Applied Chemistry* 2016, **88**(3):265-291.

7. Bjellqvist B, Hughes GJ, Pasquali C, Paquet N, Ravier F, Sanchez JC, Frutiger S, Hochstrasser D: **The focusing positions of polypeptides in immobilized pH gradients can be predicted from their amino acid sequences**. *Electrophoresis* 1993, **14**(1):1023-1031.

8. Lehninger AL: **Lehninger Principles of Biochemistry: David L. Nelson, Michael M. Cox**: Recording for the Blind & Dyslexic New York; 2004.

9. Fruton JS, Simmonds S: **General biochemistry**, vol. 76: LWW; 1953.

10. Lee B, Richards FM: **The interpretation of protein structures: estimation of static accessibility**. *Journal of molecular biology* 1971, **55**(3):379-IN374.

11. Hansch C, Leo A, Taft R: **A survey of Hammett substituent constants and resonance and field parameters**. *Chemical reviews* 1991, **91**(2):165-195.

12. Wimley WC, White SH: **Experimentally determined hydrophobicity scale for proteins at membrane interfaces**. *Nature structural biology* 1996, **3**(10):842-848.

13. Ertl P, Rohde B, Selzer P: **Fast calculation of molecular polar surface area as a sum of fragment-based contributions and its application to the prediction of drug transport properties**. *Journal of medicinal chemistry* 2000, **43**(20):3714-3717.
